# Postural impairments in unilateral and bilateral vestibulopathy

**DOI:** 10.3389/fneur.2024.1324868

**Published:** 2024-02-21

**Authors:** Julie Corre, Jean-François Cugnot, Anissa Boutabla, Samuel Cavuscens, Maurizio Ranieri, Raymond van de Berg, Robert J. Peterka, Nils Guinand, Angélica Pérez Fornos

**Affiliations:** ^1^Division of Otorhinolaryngology Head and Neck Surgery, Geneva University Hospitals, University of Geneva, Geneva, Switzerland; ^2^Division of Clinical Neurosciences, Geneva University Hospitals, Geneva, Switzerland; ^3^Division of Vestibular Disorders, Department of Otorhinolaryngology and Head and Neck Surgery, Maastricht University Medical Center, Maastricht, Netherlands; ^4^National Center for Rehabilitative Auditory Research, Veterans Administration Portland Health Care System and Department of Neurology, Oregon Health & Science University, Portland, OR, United States

**Keywords:** unilateral vestibulopathy, bilateral vestibulopathy, balance, sensory integration, posturography, vestibular

## Abstract

Chronic imbalance is a major complaint of patients suffering from bilateral vestibulopathy (BV) and is often reported by patients with chronic unilateral vestibulopathy (UV), leading to increased risk of falling. We used the Central SensoriMotor Integration (CSMI) test, which evaluates sensory integration, time delay, and motor activation contributions to standing balance control, to determine whether CSMI measures could distinguish between healthy control (HC), UV, and BV subjects and to characterize vestibular, proprioceptive, and visual contributions expressed as sensory weights. We also hypothesized that sensory weight values would be associated with the results of vestibular assessments (vestibulo ocular reflex tests and Dizziness Handicap Inventory scores). Twenty HCs, 15 UVs and 17 BVs performed three CSMI conditions evoking sway in response to pseudorandom (1) surface tilts with eyes open or, (2) surface tilts with eyes closed, and (3) visual surround tilts. Proprioceptive weights were identified in surface tilt conditions and visual weights were identified in the visual tilt condition. BVs relied significantly more on proprioception. There was no overlap in proprioceptive weights between BV and HC subjects and minimal overlap between UV and BV subjects in the eyes-closed surface-tilt condition. Additionally, visual sensory weights were greater in BVs and were similarly able to distinguish BV from HC and UV subjects. We found no significant correlations between sensory weights and the results of vestibular assessments. Sensory weights from CSMI testing could provide a useful measure for diagnosing and for objectively evaluating the effectiveness of rehabilitation efforts and future treatments designed to restore vestibular function such as hair cell regeneration and vestibular implants.

## Introduction

Loss of vestibular function in one ear, unilateral vestibulopathy (UV), or in both ears, bilateral vestibulopathy (BV), is known to affect both ascending reflexes, controlling eye movements and descending reflexes, controlling balance (1–3). Balance control has been traditionally assessed in quiet stance conditions with the availability of sensory cues of body orientation and motion manipulated in different test conditions by eye closure, stance on foam, and different stance configurations (feet together, tandem stance) (4, 5). A variation on traditional assessment uses Computerized Dynamic Posturography systems, including the Sensory Organization Test (SOT) paradigm that utilizes ‘sway-referencing’ of the stance surface and/or visual scene to greatly reduce proprioceptive and/or visual contributions to balance (6, 7). Balance performance is quantified by measuring spontaneous body sway or pass/fail (no fall/fall) performance (4, 8).

In contrast to balance assessments based on spontaneous sway measures and pass/fail performance, an alternative method was developed that used continuous small-amplitude pseudorandom rotations of the stance surface or visual surround to evoke sway responses (9, 10). In a test we now refer to as the Central SensoriMotor Integration (CSMI) test, the stimulus and sway response are processed in a manner that defines the dynamic characteristics of the stance control system. Then the parameters of a mathematical model that represents balance control as a feedback control system are adjusted to optimally account for the dynamic characteristics (5, 9–11). The identified parameters are physiologically meaningful and include the overall time delay, motor activation properties, and proprioceptive, visual, and vestibular ‘sensory weights’ which indicate the relative contribution to balance of the various sensory systems. These parameters serve as biomarkers of the balance control system.

In previous studies, sensory weight measures in vestibular deficient patients have been shown to differ from those in healthy controls (HCs). The original study using CSMI methods included 4 subjects with severe BV who were tested using stimuli that evoked antero-posterior sway (9). Results clearly demonstrated differences between 8 healthy controls (HC) and the 4 BV subjects. In BV subjects, the CSMI test performed using a surface-tilt stimulus with eyes closed provided proprioceptive sensory weight measures (*W*_prop_) that had values very close to 1.0 across tests with stimulus amplitudes ranging from 0.5° to 4° (peak-to-peak) indicating 100% reliance on proprioception and 0% reliance of vestibular cues (*W*_vest_ = 0 consistent with their vestibular loss). In contrast the *W*_prop_ values in HCs changed with stimulus amplitude ranging from ~0.7 at the 0.5° to ~0.4 at the 4° stimulus amplitude. Compared to controls, BV subjects also had higher *W*_prop_ measures on surface-tilt tests with eyes open and had higher visual weights (*W*_vis_) derived from visual-tilt tests performed on a fixed stance surface. A later study in 11 subjects with confirmed, well-compensated, complete UV found that they relied significantly more than age-matched HCs on proprioception for balance, and less on vestibular cues, even though there was no evidence for vestibular abnormality in their intact ears (12).

Another study of 11 subjects with acute peripheral vestibulopathy were tested using CSMI methods with eyes-closed surface-tilt stimuli (13). Results showed decreased *W*_vest_ compared to controls and correlation on some, but not all, clinical vestibulo-ocular reflex (VOR) measures indicative of uncompensated asymmetric vestibular function (i.e., spontaneous nystagmus examination, caloric test, rotational chair test, and head impulse test).

While studies using CSMI methods were successful in identifying abnormal sensory weights in patients with complete unilateral and severe bilateral vestibulopathy, to our knowledge there has been no direct comparison of measures from HCs to those of UV and BV patients identified in a clinical setting, where vestibular impairments may be less severe or complete, and using identical CSMI test stimuli (i.e., pseudorandom stimuli with the same amplitude and cycle duration that evoked anterior–posterior sway). It is of major importance in the field of vestibular medicine to determine whether CSMI methods facilitate the classification of vestibular disorders both for diagnosis and for the objective evaluation of novel therapies (e.g., the vestibular implant).

The goal of this study is to determine the ability of CSMI test results to distinguish between HC, UV, and BV subjects based on quantitative measures that characterize the vestibular contribution to balance and on changes in reliance on visual and proprioceptive systems that compensate for vestibular loss. A motivation for this study is to determine if specific biomarkers of postural impairments derived from CSMI testing have the potential to assess the efficacy of treatments meant to improve (e.g., vestibular rehabilitation) or restore (e.g., hair cell regeneration or prosthetic vestibular implants) vestibular function. For a biomarker to be effective as a diagnostic aid for classification of vestibular disorders and as a tool to gauge treatment or restoration effects, there should be a large difference in the biomarker between HC subjects and subjects with defective vestibular function and, ideally, there should be no overlap in biomarker values between these groups. Additionally, a biomarker represented as a continuous variable rather than a categorical variable (e.g., pass/fail or fall versus no fall) would allow for assessment of the extent of balance improvement even if balance function was not restored to normal.

Our primary focus was on sensory weight measures, but we also characterized other model-derived parameters including overall time delay and motor activation parameters, as well as measures of stimulus-evoked body and head sway, and sway variability. For sensory weight assessments we hypothesized that the *W*_prop_ value from the surface-tilt eyes-closed condition will be greatest for BV subjects (with values near 1.0), least for HCs, and with intermediate values for UVs. *W*_prop_ in the surface-tilt eyes-open condition and *W*_vis_ in the visual-tilt condition will be larger in BVs and UVs than controls, and larger in BVs than UVs with these sensory weight measures being indicative of sensory integration changes that compensate for vestibular loss. We also hypothesized that sensory weight values will be correlated with independent measures of vestibular function and with self-perceived handicap.

## Methods

### Standard protocol approvals, registrations, and participant consents

This study was conducted in accordance with the Helsinki Declaration and was approved by the local ethics committee (NAC 11–080 CER 11–129). This clinical study was registered on the SNCTP register (SNCTP000005452) and on the ClinicalTrials.gov database (NCT05246553). All participants gave written informed consent.

### Subjects

This study was conducted in 15 patients with unilateral vestibulopathy (UV) and 17 patients with bilateral vestibulopathy (BV). Results were compared to 20 gender-age matched healthy controls (HC) (see Table 1). The UV and BV group encompassed individuals over 50 years of age and suffering from uni or bilateral vestibulopathy, diagnosed following vestibular assessments described below and in agreement with the criteria consensus of the Barany Society (14). Briefly, diagnostic criteria for BV included imbalance and/or oscillopsia during walking or head movements, and a reduced bithermal caloric response (sum of bithermal maximal peak slow-phase velocity < 6°/s bilaterally) and/or bilaterally reduced video head impulse test (vHIT) gains < 0.6. Patients with unilateral vestibulopathy were recruited based on a history of sudden vertigo or imbalance with unilateral reduced vHIT gain of < 0.6 from at least one of the lateral semicircular canals and normal vHIT gain (>0.8) in the other ear. UV patients were recruited at least 6 months after acute symptoms. UV and BV patients presenting other otologic (except hearing loss), neurologic or psychiatric disease were excluded. Patients with cognitive impairments were also excluded. Moreover, other conditions such as recent hip or knee prosthesis, obesity, blindness, or polyneuropathy also automatically excluded the patient from the study. Before including a control subject, a thorough interview was conducted to ensure there was no history of balance/dizziness/neurological conditions as well as the other conditions mentioned above. All patients were recruited at the Division of Otorhinolaryngology and Head and Neck Surgery of the Geneva University Hospitals.

**Table 1:** Main demographic and anthropomorphic characteristics of the 52 patients including in this study.

	Healthy controls (HC)	Unilateral vestibulopathy (UV)	Bilateral vestibulopathy (BV)	Statistic (groups comparison) *p*-values*
Participant number (*N*)	20	15	17	–
Sex (N)				
Female	11	8	11	–
Male	9	7	6	–
Age: years, mean (SD)	59.6 (8.04)	60.8 (6)	64.8 (10.7)	0.114
Range	50–84	51–76	45–83	
Height: cm, mean (SD)	168.7 (0.07)	169.5 (0.13)	169.3 (0.1)	0.509
Weight: kg, mean (SD)	67.7 (12.7)	77.5 (18)	73.4 (12.6)	0.137
Etiology (*N*)				
Idiopathic		7	8	
Meniere’s disease		1	1	
Schwannoma		2	1	
Post labyrinthectomy		2	0	
Genetic		0	1	
Traumatic		1	1	
Meningitis		0	1	
Ototoxic		0	1	
Hydrops		0	1	
Zona		1	0	
Cyst		1	0	
Unknown		0	2	

*N*, number of subjects; SD, standard deviation; cm, centimeter; kg, kilogram, *p*-values* indicate results of Kruskal–Wallis One Way Analysis of Variance.

### Vestibular assessments

Semicircular canal function: The higher frequency range of the angular VOR was evaluated with the vHIT. Briefly, the participant was seated and wearing goggles with motion sensors that measure head movements and a camera to record eye movements (Eyeseecam, Middelfart, Denmark). A trained examiner generated unpredictable, high-velocity head impulses toward the left or the right (lateral canals) and in oblique directions that stimulated anterior and posterior canal pairs, while the participant fixated a visual target (15).

The outcome of the vHIT test was the gain of the VOR, calculated as the ratio between eye and head velocity. The gain of the vHIT is an objective measure of the efficacy of the gaze stabilization system in conditions that rely heavily on vestibular signals from the semicircular canals. To normalize the different semicircular canal dysfunction profile across BV subjects, we calculated the average gain of the VOR (averaging the gain of the six semi-circular canals). In the case of UV subjects, we calculated VOR asymmetry as it has been reported to be a good predictor of balance outcomes in this population (16) (AS; for the three canal pairs: Lateral Right vs. Lateral Left, Anterior Right vs. Posterior Left, and Posterior Right vs. Anterior Left) using the formula (Right Gain – Left Gain)/(Right Gain + Left Gain). The absolute value of these measures quantified the presence and magnitude of vestibular asymmetry.

The lower frequency range of the VOR was assessed using the caloric test. Briefly, the patient was lying in a supine position with the head elevated by 30°. Each ear was irrigated with either hot (44°C) or cold (30°) water which, respectively, excited or inhibited the lateral semicircular canals that normally result in ipsilateral or contralateral nystagmic eye movements. Absent or reduced slow phase eye velocity nystagmic responses indicated dysfunction of the horizontal semicircular canal of the side being stimulated. For the analysis of BV subjects’ caloric responses, we calculated the total response (TR), TR = (Right Cold + Left Cold + Right Warm + Left Warm) to characterize the severity of the vestibular loss. For the analysis of UV caloric responses, we calculated the Relative Vestibular Reduction (RVR), using the Jongkee’s formula as follows: ((Right Cold + Right Warm) – (Left Cold + Left Warm))/TR, with TR calculated as described above (17). All caloric response measures represent the absolute values of the peak slow phase eye velocity of caloric responses. The sign of the RVR was not of interest so the absolute values of the RVR was used to investigate the RVR relationship to other measures.

### Dizziness handicap inventory

The Dizziness handicap inventory (DHI) questionnaire was completed either in person or over the phone by all but two BV patients (who declined to answer). The DHI is a validated self-perceived handicap scale designed to evaluate the effect of dizziness and imbalance on the quality of life. The scale is based on ratings of 25 items that assess the effects of dizziness and imbalance on physical, emotional, and functional components of daily living. For each item, the subject was asked to answer ‘yes’, ‘sometimes’, or ‘no’ accounting, respectively, for 4, 2 or zero points. The total score, obtained by summing the points of each item, reflects the self-perceived handicap. Complete absence of handicap corresponds to a total score of zero, whereas 100 would be the worst self-perceived handicap (18). Self-perceived handicap is considered moderate for scores between 30 and 60, and severe for scores above 60 (19). For our study we used the total score as advised in previous studies (20, 21).

### CSMI balance test

**Figure 1:**
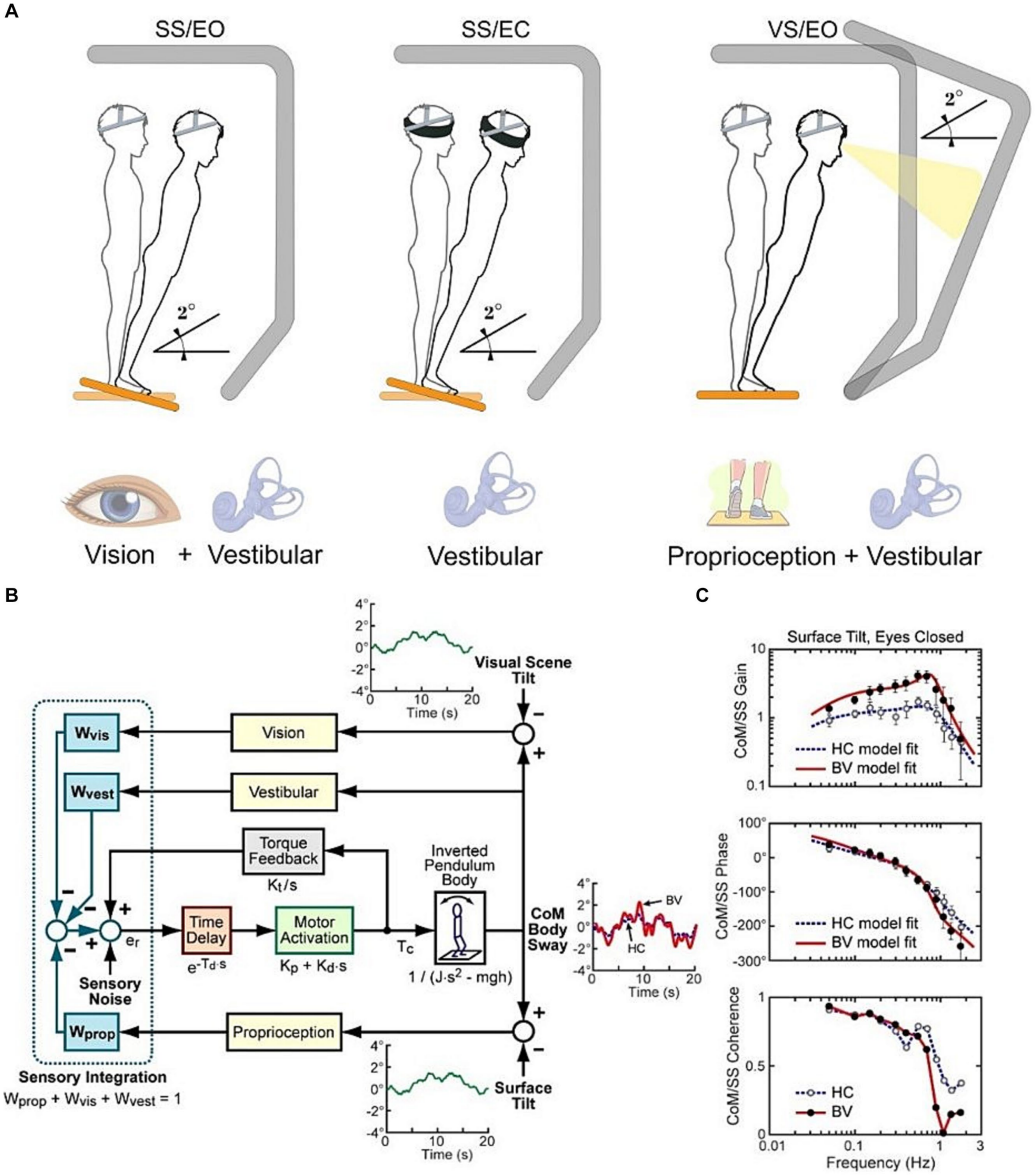
**(A)** Schematic representation of the Central Sensorimotor Integration (CSMI) test conditions. **(B)** Block diagram of CSMI feedback control model of the balance system. Each sensory system contributing to balance is represented by a “sensory weight” (W) with each weight representing the relative contribution of a sensory system to a central estimate of body motion such that the sum of all sensory weights is equal to 1.0. This central estimate in turn generates a corrective ankle torque (Tc). The model parameter values are estimated by first applying Fourier methods to calculate a Frequency Response Function **(C)** and then adjusting model parameters to optimally account for the experimental Frequency Response Function. Two example Frequency Response Functions are shown for an HC and BV subject.

Postural assessments were performed using the CSMI test (10). A schematic overview of the balance test conditions is represented in Figure 1A. CSMI testing was performed using a SMART EquiTest CRS device (Natus Medical Inc., Seattle, WA WI) programmed to deliver continuous pseudorandom support surface (SS) or visual surround (*VS*) rotations with 2° peak-to-peak amplitude that evoked antero-posterior body sway. Each four-minute test stimulus consisted of 12 repeated 20s duration cycles of the pseudorandom waveform. The subject stood either with eyes open (EO) or eyes closed (EC) and was instructed to keep arms close to their body. Up to three trials were allowed to complete a condition, but all participants completed each condition on the first trial. Participants were first familiarized with the test conditions by performing an initial 4 min warmup test consisting of 2 min of EO stance followed by 2 min of EC stance on the rotating SS. Then subjects underwent 4 min tests in three conditions: (SS/EO) surface-tilt stimuli with eyes open and visual surround fixed, (SS/EC) surface-tilt stimuli with eyes closed, and (*VS*/EO) visual surround tilt with fixed surface. Center-of-Pressure data were recorded throughout each trial from the SS force plates and were used to calculate the Center-of-Mass (CoM) displacements [see (10) for details of the calculation]. Subject anthropometric measures were used to estimate the body moment of inertia about the ankle joints and the height of the CoM above the ankle joint. The time course of the CoM sway angle relative to earth-vertical was estimated from the CoM displacement data and the estimated CoM height. Additionally, patients wore a 3-DOF Head Tracker (part of the EquiTest system) that continuously recorded angular tilt position of head movements in the Yaw, Pitch, and Roll planes. Only Pitch head pitch data were analyzed since stimuli evoked pitch-plane anterior–posterior body sway.

### CSMI analysis

Detailed description of the CSMI analysis and Matlab (The Mathworks, Natick, MA) programs to perform the analysis were previously described (10) and follow established methods for the frequency domain analysis of stimulus–response data (22, 23) with details provided in the Supplementary materials. Briefly, a mathematical feedback control model of the balance system was used to interpret the body sway evoked by the pseudorandom stimuli (Figure 1B). A frequency domain analysis of the recorded CoM angle and stimulus tilt angle was used to calculate a frequency response function (FRF) that characterized the dynamics of the balance control system (Figure 1C). The FRF was calculated by taking the ratio of the summed discrete Fourier transforms of the measured CoM sway angle of each 20-s cycle to the summed discrete Fourier transforms of each 20-s cycle of the recorded stimulus tilt angle. Only the final 11 cycles were included in the FRF calculation to eliminate transient effects at the start of the stimulus. The FRF is represented as gain and phase values across frequencies ranging from 0.05 to 1.5 Hz with its gain representing the ratio of CoM sway amplitude to stimulus amplitude and its phase representing the normalized timing of the response relative to the stimulus. The parameters of a closed-loop feedback-control model of the balance system (Figure 1B) were then adjusted to optimally account for the frequency response function data.

For each CSMI test 5 balance control parameters were estimated using constrained optimal fits to the FRF data using Matlab Optimization Toolbox ‘fmincon’ function. These included one sensory weight (*W*_prop_ on the SS/EC and SS/EO tests and *W*_vis_ on the *VS*/EO test), time delay (*Td*), motor activation stiffness (*Kp*) and damping (*Kd*) factors, and a torque feedback factor (*Kt*) that accounts for the low frequency behavior of the FRF. Each subject’s body mass (m) was measured (with 2.5% of total body mass excluded to account for the mass of the feet) and body CoM height above the ankle joint (h) and body moment of inertia (J) about the ankle joint were estimated based on anthropometric measures and total body mass (24).

The main parameters of interest were the sensory weights derived from the three test conditions which indicated the relative contribution of proprioception, vestibular, and visual information (respectively *W*_prop_, *W*_vest_, and *W*_vis_) to balance control. Because the sensory weights represent the relative contribution of a sensory system to balance control, in the SS/EC condition, where only proprioception and vestibular cues are available, the vestibular weight, *W*_vest_, is given by 1 – *W*_prop_. Therefore, in the SS/EC condition a *W*_prop_ value of 1.0 indicates that the vestibular system is making no contribution to balance. Thus, the SS/EC condition provided the most direct information about the vestibular contribution to balance while results from the other two conditions cannot separate the vestibular contribution from the contributions of proprioception and vision. Nevertheless, sensory weight measures in the SS/EO and *VS*/EO conditions can provide information on how *W*_prop_ and *W*_vis_, respectively, change when vestibular sensory cues are defective due to UV or BV losses.

Secondary parameters included: (1) a stiffness parameter (*Kp*), which is a motor activation scale factor that determined the ankle torque generated per unit of the sensory-derived estimate of the body sway angle, (2) a motor activation damping parameter (*Kd*), which determined the amount of corrective ankle torque proportional to body sway angular velocity. *Kp* and *Kd* were ‘normalized’ by dividing by *mgh* (body mass × gravity constant × height of body CoM) to allow comparison across subjects with different body anthropometrics, and (3) a time delay (*Td*) parameter that represented all the time delays in the system (sensory transduction, sensory transmission, center nervous system processing, motor nerve transmission, and muscle activation delays).

Additional time-domain measures that characterized CSMI test responses included (1) calculation of the zero-meaned, root mean square (RMS) value of the cycle-averaged, stimulus-evoked CoM angle and head pitch sway angle with the cycle average occurring across the last 11 of the 12 stimulus cycles, and (2) the RMS value of ‘remnant sway’ of both the CoM and head pitch sway angle. The remnant sway provided a measure of sway variability that was not accounted for by mean values of the stimulus-evoked sway. Examples of cycle-averaged CoM sway for an HC and BV subject are shown in Figure 1B.

Finally, to test the effect of both the sensory weight and the stiffness factor *Kp* on the stimulus sensitivity we plotted the stimulus-evoked sway against W*(*Kp*/(*Kp*-*mgh*)) value which includes the contribution of the sensory weight and the influence of the stiffness *Kp* on overall sensitivity (25).

### Statistical analysis

Groups’ demographic differences were assessed based on age, height, and mass with Kruskal–Wallis One Way Analysis of Variance, and also for DHI outcomes with a Student’s *t*-test.

The analyses of the parameters from the CSMI model were carried out with SigmaPlot 14 (Systat Software, San Jose, CA, USA). The first step in the analysis was to look at the distribution (central tendency and dispersion) as well as the validity of the normality assumption with statistical tests (Shapiro–Wilk). This allowed choice of the appropriate test variants, i.e., parametric (One Way ANOVA) or not (Kruskall–Wallis). The Hedge’s *G* value, which represents an effect size measure evaluating the difference between means relative to the standard deviation, was also calculated (26).

For all correlation analyses (sensory weights versus vHIT asymmetry for UV subjects and sensory weights versus average vHIT gain for BV subjects; sensory weights versus total caloric response for BV subjects and sensory weights versus relative vestibular reduction for UV subjects and finally sensory weights versus DHI score and vHIT outcomes versus DHI score) the Pearson product–moment correlation coefficient was determined.

For all statistical tests, the significance was defined as a *p* value less than 0.05.

## Results

After presenting demographic data we summarize vestibular assessments from conventional clinical vestibular tests and DHI measures. Then CSMI time domain measures of stimulus-evoked CoM body and head sway are described along with remnant sway measures (sway variability not accounted for by stimulus-evoked sway) followed by CSMI parameters derived from a model-based assessment of stimulus-evoked balance responses. Finally, various relationships among clinical vestibular tests, CSMI measures, and DHI are presented along with results demonstrating how CSMI sensory weights and stiffness measures are related to stimulus-evoked sway.

### Demographic and anthropometric data

Fifty-two participants were included in this study and split into the three following groups, healthy control subjects (HC, *n* = 20, 11 females and 9 males) and patients with either unilateral or bilateral vestibulopathy. Demographic and anthropomorphic data are presented in Table 1. For these descriptors there was no significant difference in age, height, or weight between the three groups (*p* > 0.05).

### Vestibular assessments

The outcomes of vestibular assessments are presented in Table 2.

**Table 2:** Vestibular assessments.

	Unilateral vestibulopathy (UV)	Bilateral vestibulopathy (BV)
vHIT assessments		
Completed tests	15	17
vHIT asymmetry LR vs. LL: mean, (SD)	0.61 (0.24)	
vHIT asymmetry AR vs. PL: mean, (SD)	0.17 (0.19)	
vHIT asymmetry PR vs. AL: mean, (SD)	0.15 (0.12)	
vHIT average gain: mean, (SD)		0.19 (0.26)
Caloric assessments		
Completed tests	13	15
Relative vestibular reduction % (mean, SD)	64.4 (23.6)	
Average response: deg/s, mean, (SD)		7.41 (7.36)
Vestibular symptoms assessment		
Completed questionnaire	15	15
DHI score: mean, (SD)	29.2 (22.8)	43.9 (20.1)

vHIT, video head impulse test; LR, lateral right; LL, lateral left; AR, anterior right; AL, anterior left; PR, posterior right; PL, posterior left. For BV subjects, average vHIT gain of head impulses in all six directions. DHI, dizziness handicap inventory; SD, standard deviation.

In BV subjects, the average vHIT gain across all 6 directions of head impulses was 0.19 ± 0.26 (mean ± standard deviation - SD), consistent with the diagnostic criteria for BV (14). BV caloric responses showed a low average total response (TR = 7.41 ± 7.36 deg./s), consistent with bilaterally reduced lateral canal function.

In UV subjects, the vHIT results showed large lateral canal asymmetry (average Lateral Right vs. Lateral Left asymmetry = 0.61). Twenty-four of the 30 vertical canal asymmetry measures (Anterior Right vs. Posterior Left and Posterior Right vs. Anterior Left) from the 15 UV subjects had values less than 0.3 (i.e., within normal limits) and only one UV subject had a larger vertical than lateral canal vHIT asymmetry. These results indicate that lateral canals were generally more affected than vertical canals in our UV population. In UV subjects we found an average absolute value of RVR of 64.4% (SD: 23.6%) consistent with unilateral loss of lateral canal function.

**Figure 2:**
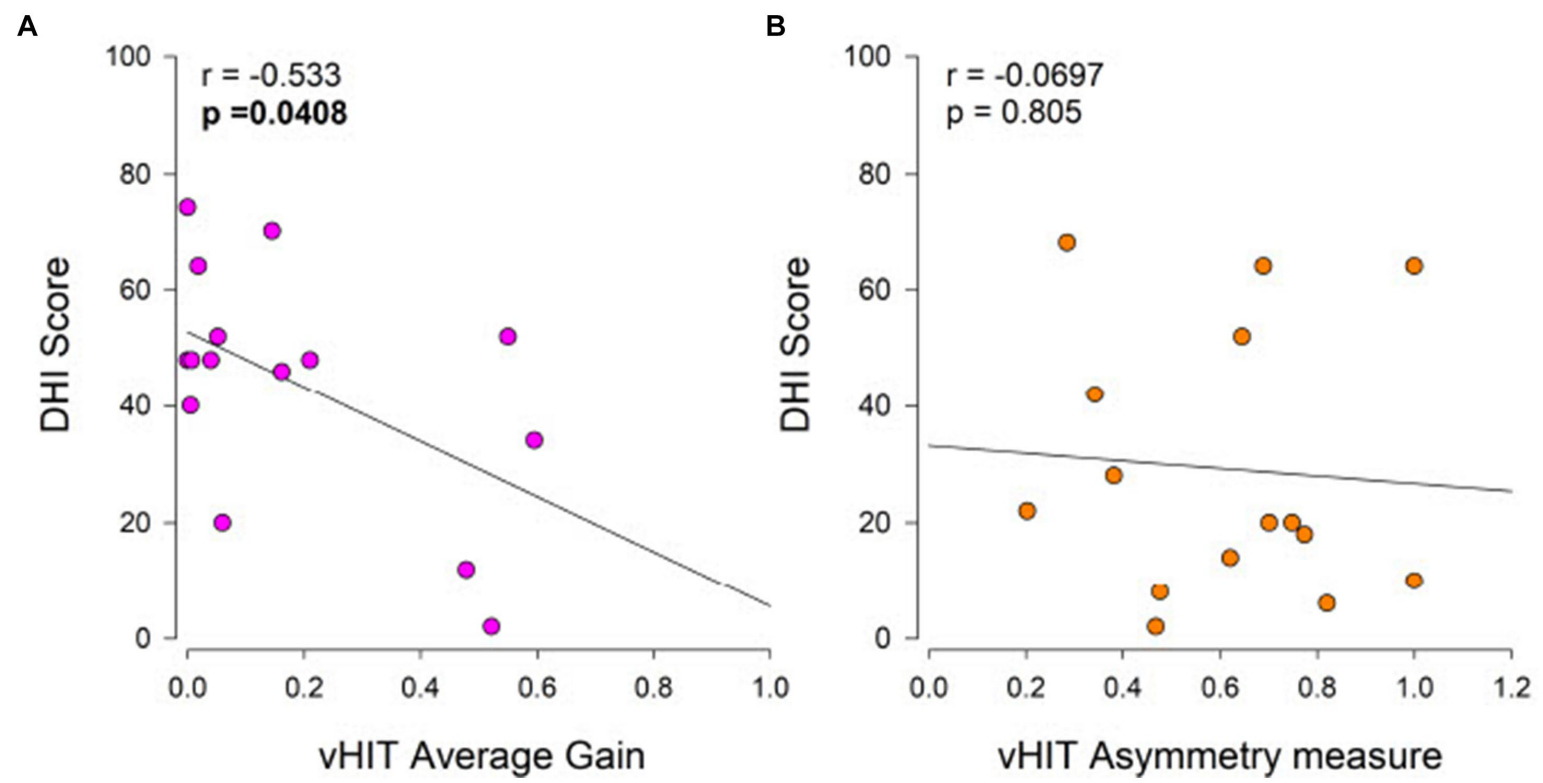
Relationship of Dizziness Handicap Inventory (DHI) to vHIT average gain in BV subjects **(A)** and to vHIT lateral canal asymmetry in UV subjects **(B)**. Self-perceived handicap is considered moderate for DHI scores between 30 and 60, and severe for DHI scores above 60. The Pearson product–moment correlation coefficient (*r*) and associated *p*-values are shown.

The average DHI scores for BV and UV subjects were 43.9 and 29.2, respectively, which are indicative of perceived disability that is approximately in the moderate range. There was large variability among DHI scores for both BV and UV subjects and there was no significant difference between the means (Student’s *t*-test, *p* = 0.072). The DHI score and the vHIT average gain were significantly correlated for BV subjects (Pearson product–moment correlation = −0.533, *p* = 0.041) meaning that those with lower vHIT gains had higher DHI scores (Figure 2A) but we did not find a significant correlation for UV subjects (Figure 2B).

### CSMI test assessments – time domain sway measures

**Figure 3:**
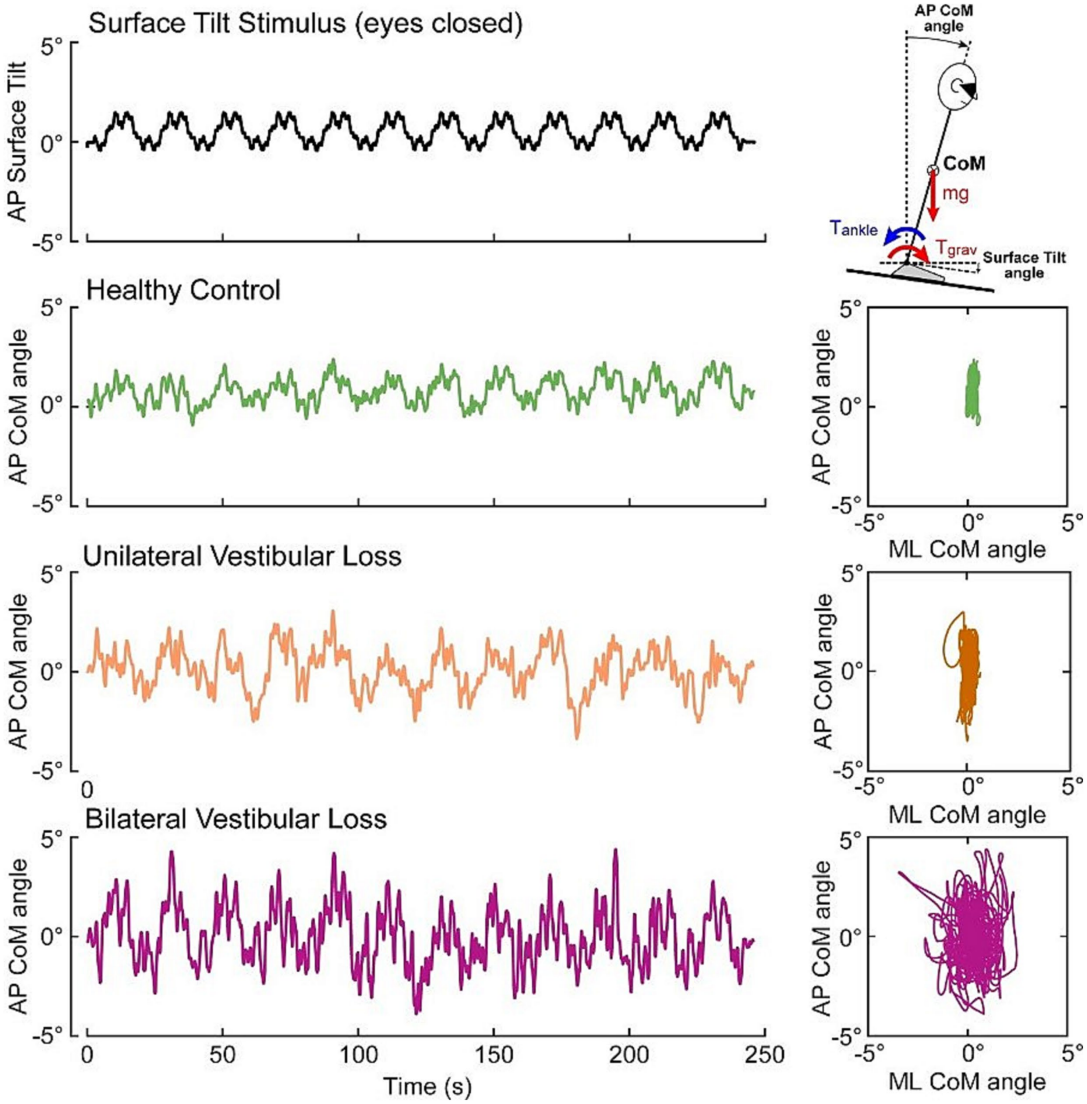
Example data from an eyes closed CSMI surface tilt stimulus (black trace) evoking center of mass (CoM) sway in an individual HC subject (green trace), a UV subject (orange trace), and a BV subject (purple trace). Right panels display anterior–posterior (AP) sway in relation to the medial-lateral (ML) sway.

**Figure 4:**
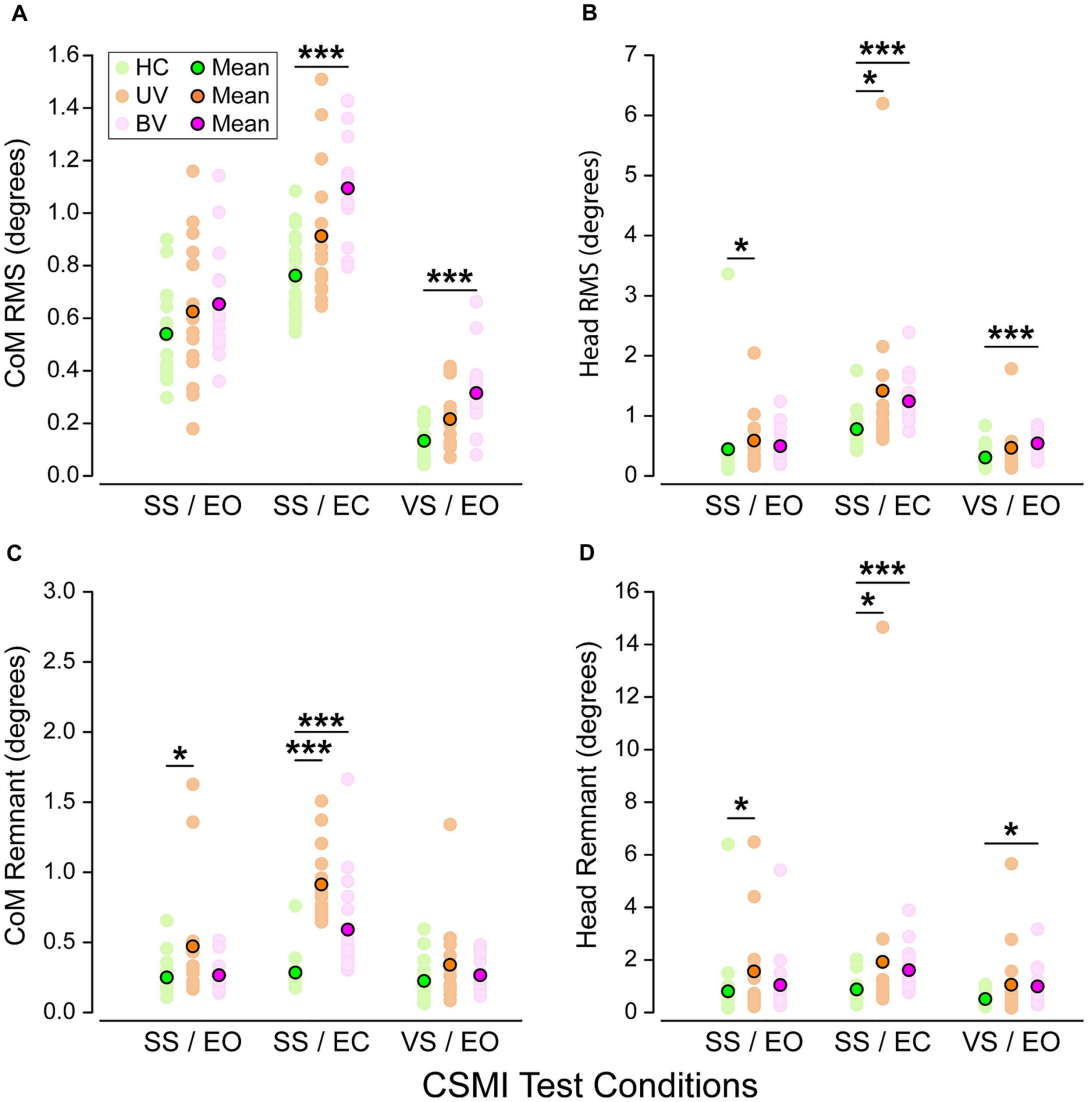
Scatter plots of root mean square (RMS) values of **(A)** stimulus-evoked Center of Mass (CoM) sway, **(B)** stimulus-evoked head sway, **(C)** remnant CoM sway, and **(D)** remnant head sway in healthy controls (HC, green), unilateral vestibular loss (UV, orange) and bilateral vestibular loss (BV, purple) groups and for the three CSMI test conditions. Pastel dots represent individual subject values while bright ones are group mean values. Stars and black horizontal lines indicate significant differences between groups (*p < 0.05, **p < 0.01, ***p < 0.001).

Example CoM sway traces from single HC, UV, and BV subjects performing a CSMI eyes-closed surface tilt test are shown in Figure 3. CSMI assessments include measures of stimulus-evoked CoM and head sway, remnant CoM and head sway, and CSMI model parameters. The individual and mean RMS values of evoked sway and remnant sway for the 3 groups and 3 CSMI test conditions are shown in Figure 4. Numerical results as well as post-hoc comparison statistics between test conditions are also presented in Supplementary Tables S1, S3, respectively.

#### Stimulus-evoked CoM sway

Across the three CSMI conditions the mean RMS value of stimulus-evoked CoM sway was smallest in the *VS*/EO condition, larger in the SS/EO condition, and largest in the SS/EC condition (Figure 4A). *Post-hoc* comparisons showed significant differences between all paired test conditions. Within each CSMI condition, the mean RMS value of stimulus-evoked CoM sway was lowest for HCs, larger for UVs, and largest for BVs. However, there was considerable overlap in individual RMS values of the three groups in each condition such that significant between-group differences were only identified between HCs and BVs in the SS/EC and the *VS*/EO conditions.

#### Stimulus-evoked head sway

Across the three CSMI conditions the mean RMS value of stimulus-evoked head sway was smallest in the *VS*/EO condition, larger in the SS/EO condition, and largest in the SS/EC condition (Figure 4B). *Post-hoc* comparisons showed significant differences between the SS/EO and SS/EC conditions as well as between SS/EC and *VS*/EO. For the SS/EO and SS/EC conditions, the mean RMS value of stimulus-evoked head sway was lowest for HCs, larger for BVs, and largest for UVs. For the *VS*/EO condition BVs had the largest head sway. Significant between-group differences were identified in all conditions. Specifically, in the SS/EO condition there were significant differences between HCs and UVs, in the SS/EC condition between HCs and BVs as well as HCs and UVs, and in the *VS*/EO condition between HCs and BVs. However, there was considerable overlap in individual RMS values of the three groups and one outlier HC subject in the SS/EO condition and one outlier among UV subjects in all test conditions.

#### CoM remnant sway

Across the three CSMI conditions the mean RMS value of the remnant CoM sway was smallest in the *VS*/EO condition, larger in the SS/EO condition, and largest in the SS/EC condition (Figure 4C). Post-hoc comparisons showed significant differences between SS/EO and SS/EC as well as between the SS/EC and *VS*/EO test conditions. Remnant sway quantifies the variability of the CoM sway that was not accounted for by the stimulus-evoked sway. Thus, finding greater sway variability in the SS/EC condition was expected because of one fewer sensory system contributing to an internal estimate of body orientation than in the other two conditions. Within each CSMI condition, the mean RMS value of CoM remnant sway was lowest for HCs, larger for BVs, and largest for UVs. There was considerable overlap in individual RMS CoM remnant sway values in the three groups in each condition. However, significant between-group differences were identified in the SS/EO condition between HCs and UVs and in the SS/EC condition between HCs and UVs as well as between HCs and BVs.

#### Head remnant sway

Across the three CSMI conditions the mean RMS value of remnant head sway was smallest in the *VS*/EO condition, larger in the SS/EO condition, and largest in the SS/EC condition (Figure 4D). Post-hoc comparisons showed significant differences between SS/EC and *VS*/EO and between SS/EC and SS/EO conditions. Within each CSMI condition, the mean RMS value of head remnant sway was lowest for HCs, larger for BVs, and largest for UVs. Significant between-group differences were identified in all conditions. Specifically, differences were found in the SS/EO condition between HCs and UVs, in the SS/EC condition between HCs and BVs and between HCs and UVs, and in the *VS*/EO condition between HCs and BVs. There was considerable overlap in individual RMS values of the three groups and one outlier HC subject in the SS/EO condition and UV outliers in all test conditions.

### CSMI test assessments – balance control parameters

**Figure 5:**
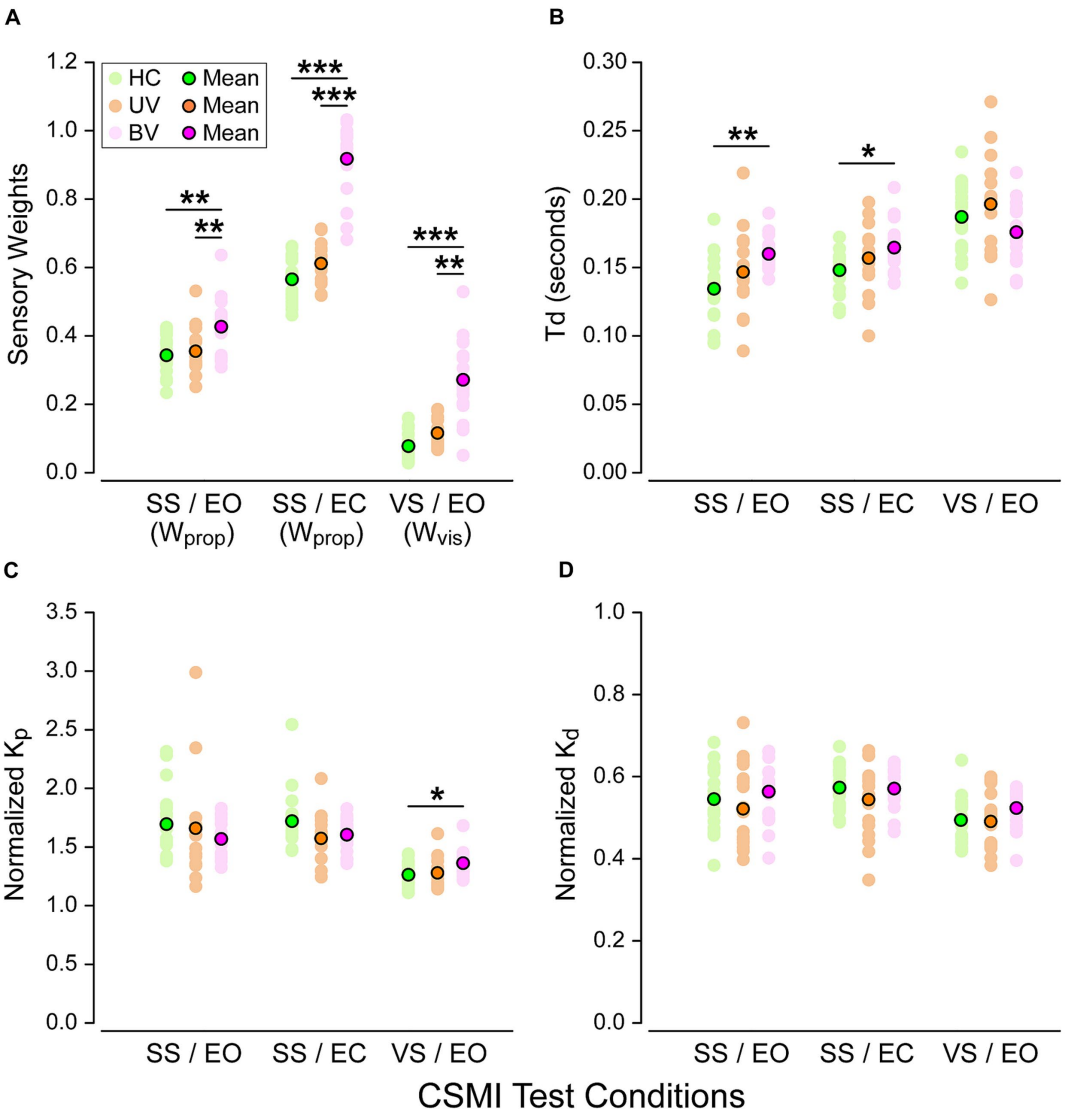
Balance control model parameters across the three CSMI testing conditions in healthy controls (HC, green), unilateral vestibulopathy (UV, orange) and bilateral vestibulopathy (BV, purple) groups. Plots display **(A)** sensory weights (*W*_prop_ or *W*_vis_ depending on the test condition), **(B)** time delays *T*_d_, **(C)** normalized *K*_p_, and **(D)** normalized *K*_d_. Stars and black horizontal lines indicate significant differences between groups (*p < 0.05, **p < 0.01, ***p < 0.001).

The individual and mean CSMI parameters for the 3 groups and 3 CSMI test conditions are shown in Figure 5. Numerical results as well as post-hoc comparisons between test conditions are also presented in Supplementary Tables S2, S3, respectively.

#### Sensory weights (*W*_prop_ and *W*_vis_)

The model-based interpretation of stimulus-evoked sway included estimates of *W*_prop_ in the SS/EO and SS/EC conditions and *W*_vis_ in the *VS*/EO condition.

*Post-hoc* comparisons showed significant differences between all paired test conditions. Moreover, in the three test conditions, we found that sensory weights in BV subjects were consistently larger than those in HC and UV subjects (Figure 5A). The differences were significant between both BV and HC and between BV and UV subjects. In particular in the SS/EC condition *W*_prop_ was 62% larger compared to HC and 50% compared to UV subjects. *W*_prop_ was close to 1.0 (and therefore *W*_vest_ close to zero) for 13 of the 17 BV subjects indicating essentially no ability to use vestibular information for balance, consistent with their BV diagnosis based on clinical vestibular tests. Additionally, there was no overlap in individual *W*_prop_ measures in BV and HC subjects, and only minimal overlap between BV and UV values. However, across all three test conditions sensory weight measures in UV subjects were only slightly higher (8%) than in HC subjects and none of these differences were significant. This minimal difference between HC and UV in the SS/EC condition indicates that the UV loss in this particular cohort of patients resulted in only a small decrease in their ability to use vestibular information for balance.

In the *VS*/EO condition, BV subjects relied significantly more on visual cues than both HC and UV subjects (respectively 247% and 126% larger visual weights). Although there was a large separation between mean values of *W*_vis_ in BV compared to HC and UV subjects, there was some overlap in individual *W*_vis_ measures in the HC and BV subjects which did not occur in the SS/EC condition. Finally, in the SS/EO condition we also observed significant differences regarding reliance on proprioceptive cues, with a significant increased reliance in BV subjects compared to both UV and HC subjects (respectively *W*_prop_ 24% and 20% larger). However, UV and HC subjects had similar reliance on proprioceptive cues (only 3% larger for UV subjects).

#### Time delay

Time delay (*Td*) results are shown in Figure 5B. Post-hoc comparisons showed significant differences between the *VS*/EO and SS/EO conditions as well as between *VS*/EO and SS/EC. In the SS/EO and SS/EC conditions time delay was shorter in HC, longer in UV, and longest in BV subjects. Time delay differences between BV and HC subjects were significant in the SS/EO and SS/EC condition with 19% and 11%, respectively, longer delays in BV subjects. We did not observe any significant differences between groups in the *VS*/EO condition (*p* = 0.131).

#### Motor activation parameters: normalized stiffness (*Kp*/*mgh*) and normalized damping (*Kd*/*mgh*)

The motor activation parameters characterize the transformation from the internal sensory-derived estimate of body orientation and motion to the corrective ankle torque needed to resist gravity and stabilize upright stance. These parameters can affect the sensitivity of sway responses to internal and external perturbations to balance (25). For both parameters, post-hoc comparisons showed significant differences between the *VS*/EO and SS/EO condition as well as between the *VS*/EO and SS/EC ones. However, our results showed only minimal differences between the three groups (Figures 5C,D) in the three test conditions. Indeed, only the *VS*/EO condition revealed significant differences in normalized stiffness between HC and BV subjects (*p* = 0.027).

### Relations between sensory weights, clinical vestibular measures, and DHI

**Figure 6:**
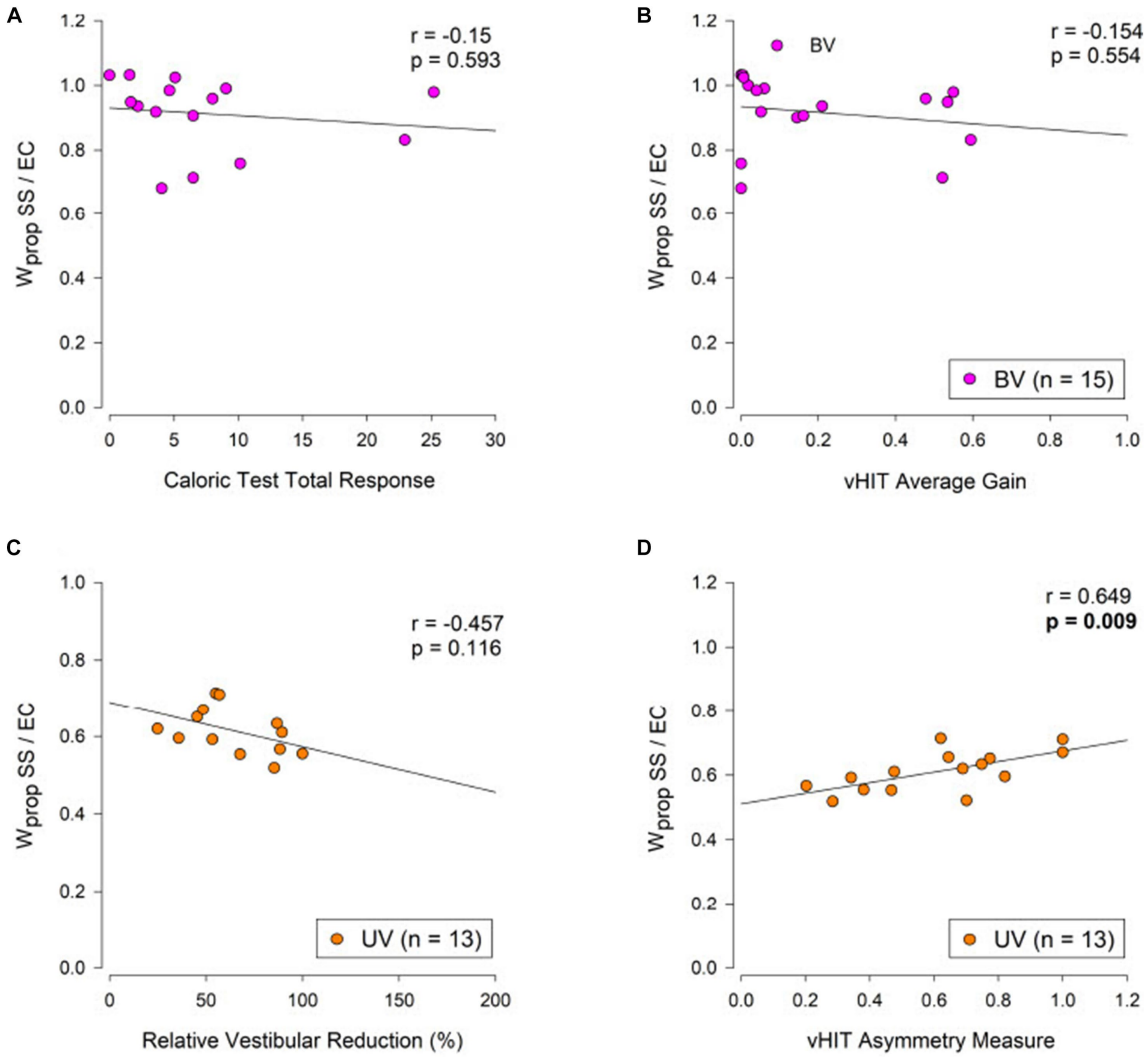
Relation of caloric test and vHIT measures to proprioceptive sensory weight measures from the eyes-closed surface tilt (SS/EC) condition in bilateral vestibulopathy (BV, purple points, **A,B**) and unilateral vestibulopathy (UV, orange points, **C**,**D**) subjects. The Pearson product–moment correlation coefficient (*r*) and corresponding *p*-values are shown.

We observed very small trends in the expected directions, but these relations were not at all close to being significant. However, we observed two distinct functional clusters (Figures 6A,B). Correlations between caloric TR and vHIT average gain with sensory weight measures in the SS/EO and *VS*/EO conditions were also calculated with no significant correlations found (Table 3).

**Table 3:** Relations between sensory weights, clinical vestibular measures, and DHI.

	Groups											
	UV						BV					
	Sensory Weight, Condition						Sensory Weight, Condition					
	*W*_prop_ SS/EO		*W*_prop_ SS/EC		W_vis_ *VS*/EO		W_prop_ SS/EO		W_prop_ SS/EC		W_vis_ *VS*/EO	
	Pearson correlation	*p-*value	Pearson correlation	*p-*value	Pearson correlation	*p-*value	Pearson correlation	*p-*value	Pearson correlation	*p-*value	Pearson correlation	*p-*value
vHIT test												
Asymmetry LR vs. LL	−0.136	0.629	0.649	**0.009**	0.203	0.469	**–**	**–**	**-**	**-**	**-**	-
Average gain	–	–	–	–	–	–	0.052	0.844	−0.154	0.554	0.082	0.754
Caloric test												
Relative vestibular reduction	−0.089	0.78	−0.457	0.116	−0.229	0.451	–	–	–	–	–	–
Total response	–	–	–	–	–	–	0.223	0.424	−0.15	0.593	0.04	0.888
DHI score												
	0.119	0.697	−0.188	0.538	0.641	**0.018**	0.274	0.324	0.356	0.193	0.036	0.899

SS/EO, surface stimulus eyes open; SS/EC, surface stimulus eyes closed; VS/EO, visual stimulus eyes open; W_prop_, proprioceptive weight; W_vis_, visual weight; vHIT, video head impulse test; LR, lateral right; LL, lateral left. For BV subjects, average vHIT gain of head impulses in all six directions. DHI, dizziness handicap inventory. The Pearson product–moment correlation coefficient (*r*) between sensory weight values and vestibular assessments outcomes was determined and the significance was defined as a *p-*value (*p*) inferior to 0.05. Bolded outcomes indicate a significant group difference.

For UV subjects we anticipated that *W*_prop_ in the SS/EC condition would be correlated with the level of vestibular asymmetry as quantified by the caloric RVR measure and the vHIT asymmetry measure with the expectation that *W*_prop_ would be larger in subjects with greater vestibular asymmetry. Results showed no correlation of SS/EC *W*_prop_ with caloric RVR but did show the expected significant increase in *W*_prop_ with the vHIT asymmetry measure (Figures 6C,D). Correlations between caloric RVR and vHIT gain asymmetry with sensory weight measures in the SS/EO and *VS*/EO conditions were also calculated with no significant correlations found (Table 3).

Potential relationships between perceived disability quantified by the DHI score and sensory weights in the 3 CSMI test conditions were investigated in UV and BV subjects with correlation shown in Table 3. There were no significant correlations for BV subjects. For UV subjects the only significant correlation with DHI was found between *W*_vis_ in the *VS*/EO condition where larger (worse) DHI scores were associated with larger *W*_vis_ values (Supplementary Figure S2).

### Relation between sensory weight values and CoM sway measures

**Figure 7:**
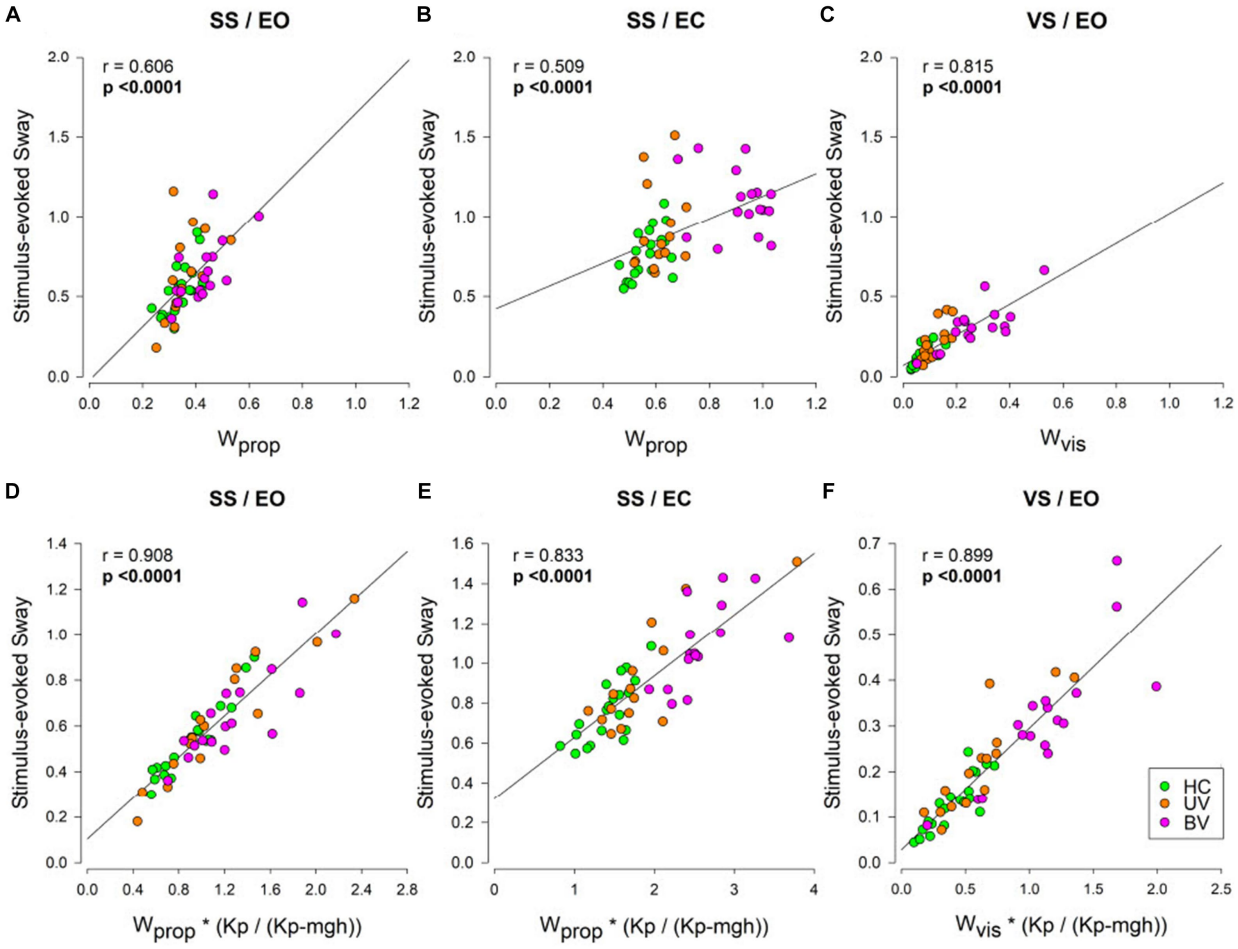
CSMI parameters accounting for the variation in RMS values of stimulus-evoked sway. CoM sway measure versus sensory weight values across the three testing conditions in healthy controls (HC, green), unilateral vestibulopathy (UV, orange), and bilateral vestibulopathy (BV, purple) groups. In panels **(A–C)** stimulus-evoked sway measures are plotted against the CSMI sensory weight parameters, while in Panel **(D–F)** stimulus-evoked sway measures are plotted against the product of the sensory weight parameters and the stiffness-related sway amplification factor Kp/(Kp – mgh). Panels **(A,D)** show *W*_prop_ for the SS/EO condition, panels **(B,E)** show *W*_prop_ for the SS/EC condition, and panels **(C,F)** show *W*_vis_ for the *VS*/EO condition. The Pearson product–moment correlation coefficient (*r*) are show with associated *p*-values. Bold *p*-values indicate statistically significant correlations.

Across all three test conditions we observed significant correlations between the stimulus-evoked CoM sway and the respective sensory weights (Figures 7A–C). In the SS/EO condition (Figure 7A) the *W*_prop_ accounted for 37% of the variance (*r*^2^ = 0.37), and around one quarter of the variance in the SS/EC condition (Figure 7B) (*r*^2^ = 0.26), while in the *VS*/EO condition (Figure 7C) the *W*_vis_ accounted for two thirds of the variance (*r*^2^ = 0.66). Stimulus sensitivity was not only affected by the sensory weight but also by the stiffness factor *Kp*. Although *Kp* did not differ between groups the variation observed for that parameter within each condition could still contribute to an individual’s stimulus-evoked sway. The results presented in Figures 7D–F showed that the W*(*Kp*/(*Kp*-*mgh*)) value accounted for more of the variance than the sensory weight alone. Indeed, in the SS/EO condition (Figure 7D) *W*_prop_*(*Kp*/(*Kp*-mgh)) accounted for 82.5% of the variance (*r*^2^ =0.825), 69.4% in the SS/EC condition (Figure 7E) (*r*^2^ =0.694), while in the *VS*/EO condition (Figure 7F) *W*_vis_*(*Kp*/(*Kp*-*mgh*)) accounted 80.8% of the variance (*r*^2^ = 0.808).

## Discussion

The goal of this study was to determine whether results from CSMI tests were able to distinguish among HC, UV, and BV subjects with the vestibular status of UV and BV subjects determined from clinical caloric and vHIT tests. The motivation was not just to demonstrate significant differences between group mean values, but to determine whether any CSMI measure could reliably demonstrate the presence of vestibular dysfunction in an individual subject and could distinguish between UV and BV dysfunction. The existence of such a measure could then contribute to the evaluation of the effectiveness of rehabilitation efforts and of future treatments designed to restore vestibular function such as hair cell regeneration and vestibular implants.

### Stimulus evoked sway and sway variability measures

Results from CSMI tests included time-domain measures characterizing the RMS value of the average stimulus-evoked CoM sway and head sway, as well as the variability of CoM and head sway (RMS values of remnant sway) (Figure 4).

We expected that greater loss of vestibular function would result in greater stimulus-evoked CoM and head sway. On average, this was confirmed in the RMS CoM sway in all CSMI test conditions with the greatest mean stimulus-evoked sway occurring in BV subjects, a lower mean sway in UV subjects, and the least mean sway in HC subjects (Figure 4A). However, there was considerable overlap of individual values across the three groups in each test condition. While group differences between HC and BV were statistically significant in the SS/EC and *VS*/EO tests a number of BV subjects had RMS sway measures that overlapped with those of HC subjects. The least separation between groups occurred in the SS/EO condition consistent with the possibility that vestibular deficient subjects could have reduced their sensitivity to surface-tilt balance disturbances by relying more on the orientationally accurate visual cues available in that condition. This compensatory effect effectively masked differences between groups in this test condition.

RMS stimulus-evoked head sway results (Figure 4B) were similar to CoM sway results in that the head sways were generally larger in UV and BV subjects than in HCs and there were statically significant differences between HC and BV subjects in SS/EC and *VS*/EO conditions but not in the SS/EO condition. Results were also similar to CoM sway results in that there was considerable overlap in individual measures from the three groups.

The RMS values of remnant CoM and head sway quantified the sway variability that was not accounted for by the mean stimulus-evoked sway. This variability is somewhat analogous to spontaneous sway measures where greater sway has been associated with changes in availability of accurate sensory information (i.e., stance on foam, eyes closed stance, sensory deficits, neurological diseases) (4, 5, 25, 27). Thus, the expectation was that remnant CoM and head sway would be greatest in BV subjects, less in UV, and least in HC. While mean values of CoM and head remnant sway were larger in UV and BV compared to HC subjects, not all these differences were significant (Figures 4C,D). Furthermore, and in contrast to expectations, remnant sways were larger in UV than BV subjects with individual outlying UV subject measures influencing the mean remnant sway values. The clearest between-group differences were in the SS/EC condition consistent with generally greater variability occurring in this condition where the fewest sensory systems were contributing to balance. As with the stimulus-evoked sway measures there was considerable overlap among individual results from the three groups in each test condition. A single exception was in the SS/EC condition for the CoM sway remnant. In this condition only one outlying HC value overlapped with the range of UV CoM remnant sway values. However, the BV values in this condition overlapped with both the HC and UV values. We have no explanation for the higher remnant sway variability in UV compared to BV groups.

Overall, due to the large overlaps in individual stimulus-evoked and remnant sway measures (Figure 4), neither provided for satisfactory classification of UV or BV deficits based on results from individual subjects. However, these measures could still be potentially useful for tracking treatment-related changes in an individual patient’s balance control.

### CSMI model parameter measures

The major differences between groups occurred among the sensory weight measures with relatively small and mostly non-significant between group differences in the other parameters (Figure 5). Therefore, we focus on understanding results pertaining to the sensory weights.

In the original development of the CSMI balance test four subjects with severe BV loss were tested in six conditions (10). In the three test conditions also used in the present study, all three showed a pattern of sensory weight measures similar to the original study. Specifically, in the 2002 study, for stimuli with 2 degrees peak-to-peak amplitudes, the mean *W*_prop_ values in the SS/EC and SS/EO conditions were 0.986 (0.021 SD) and 0.491 (0.118 SD), respectively, and the mean *W*_vis_ value in the *VS*/EO condition was 0.384 (0.033 SD). These mean sensory weights are all slightly greater than the mean values in the current study (Supplementary Table S2). A possible explanation could be that the four BV subjects in the earlier study had more severe vestibular loss than at least some of the BV subjects in the current study. Additionally, the pseudorandom stimuli used in the two studies were not identical. Despite these differences, the previous and current studies revealed very large differences in sensory weight values between HC and BV subjects particularly in the SS/EC and *VS*/EO conditions (Figure 5A). Specifically, in the current study there was no overlap between HC and BV subjects in individual W_prop_ measures in the SS/EC condition and only three BV subjects had W_vis_ measures within the range of HC ones in the *VS*/EO condition. Results from the earlier study (10) suggest that use of a larger amplitude visual stimulus could produce W_vis_ measures that further differentiate between HC and BV subjects. Specifically, the earlier study demonstrated that subjects with normal sensory function decrease the sensory weighting with increasing stimulus amplitude while this did not occur or occurred to a lesser extent in the four BV subjects. Thus, use of a 4 degrees peak-to-peak visual stimulus, which likely would not cause loss of balance in BV subjects, could widen the difference between *W*_vis_ values from HC and BV subjects.

A previous study that used CSMI related test methods to investigate differences in balance control between subjects with complete UV loss and age-matched controls demonstrated that *W*_prop_ measures in an SS/EC condition could discriminate between HC and UV subjects to a much greater extent than results from the current study (12). While results are not directly comparable (the previous study used different pseudorandom stimuli that evoked frontal plane sway rather than anterior–posterior sway) UV subjects in the previous study had complete UV loss while vHIT testing of 13 of the UV subjects in the current study showed limited evidence of asymmetry in the vertical semicircular canals, which with the otolith organs (especially the utricule), are important contributors to standing balance control. Thus, we would not expect that balance in our UV subjects, presenting mainly dysfunction of the lateral semicircular canal, would be affected as much as balance in UV subjects with complete loss in one ear. That is, the incomplete vestibular loss in many of our UV subject could explain the relative similarity of sensory weight measures in HC and UV subjects in our study.

Overall, results using sensory weights (Figure 5A) showed much better differentiation between BV and HC subjects than did RMS sway measures (Figure 4A) based on the absence in overlap of individual sensory weights between the two groups in the SS/EC condition and minimal overlap in the *VS*/EO condition. Additionally, Hedge’s G measures comparing BV and HC results were larger for sensory weight measures than for RMS sway measures in all CSMI conditions (Supplementary Figure S1).

### CSMI parameters accounting for the variation in RMS values of stimulus-evoked sway

The larger stimulus-evoked sways (both CoM and Head) observed in BV subjects compared to HCs indicated an increased sensitivity to the presented stimulus but were less useful than sensory weights in distinguishing between HC and BV subjects. The CSMI parameters provide insight into why sensory weight measures were better able to distinguish HC from BV subjects than were stimulus-evoked sway measures.

A recent study investigating postural deficits in people with chronic mild traumatic brain injury (25) showed that sensitivity was affected by both sensory weights and by the stiffness factor *Kp*. In the current study, although mean *Kp* did not differ between groups there was still variation in *Kp* within each group that would be expected to influence an individual’s stimulus-evoked sway. The results presented in Figure 7 showed that, even though the sensory weights accounted for a significant part of the variance in the relationship (Figures 7A–C), the stiffness factor *Kp* also affected the magnitude of stimulus-evoked sway (Figures 7D–F). Thus, the added influence of *Kp* likely increased the variability of the stimulus-evoked sway measures resulting in sway measures (Figure 4A) being poorer at differentiating between groups than sensory weight measures (Figure 5A).

### Relation between sensory weight measures and clinical measures of vestibular function

We hypothesized that sensory weight values would be correlated with independent clinical test measures of vestibular function (13). The general expectation was that, among BV subjects, clinical measures indicative of greater levels of bilateral vestibular loss (caloric total response and vHIT average gain) would be associated with greater reliance on sensory systems other than the vestibular system for balance control. In particular, since the SS/EC condition provides a measure *W*_vest_ = 1 – *W*_prop_, we anticipated that *W*_vest_ would be smaller (and *W*_prop_ larger) in BV subjects with smaller caloric TR measures and, similarly, *W*_prop_ would be larger in BV subjects with lower values of vHIT average gain. For UV subjects, clinical measures indicative of greater levels of asymmetric vestibular function (caloric reduced vestibular response and vHIT asymmetry) also would be associated with greater reliance on sensory systems other than the vestibular system for balance control. That is, larger vestibular asymmetry measures indicate greater loss of vestibular function in one ear with the net effect that the vestibular orientation information available to the brain is less reliable (i.e., noisier with reduced precision) prompting a reweighting toward increased reliance on sensory cues other than vestibular (28, 29). Additionally, as with BV subjects, the expectation for UV subjects was that the SS/EC CSMI test condition would be the most likely condition to reveal correlations since reduced reliance on vestibular information would be directly revealed by an increased reliance on proprioception.

The above expectations were almost entirely not confirmed statistically although the trends were in the expected directions for both BV and UV subjects in the SS/EC condition (Table 3 and Figure 6). The single exception with statistical significance was the relation between *W*_prop_ and vHIT lateral canal asymmetry for UV subjects (Figure 6D). Other than this relationship which showed a reasonably high correlation coefficient of 0.649, correlations were quite low with trends often not consistent with the expected directions in the SS/EO and *VS*/EO conditions.

A previous study investigated the relationship between results from a similar SS/EC CSMI test and various clinical vestibular test measures, including caloric test, rotation test, and vHIT (13). Their subjects were categorized as having acute vestibular dysfunction that was unspecified as being unilateral or bilateral. That study found that their subjects relied less on vestibular function for balance control. This could be considered consistent with our results that showed larger *W*_prop_, and therefore lower *W*_vest_ (Figure 5A) in UV and BV subjects although we found no significant difference between our UV and HC subjects. However, none of our UV subjects were in the acute phase of their vestibular loss. Consistent with our results the previous study similarly found only weak or absent relationships between *W*_vest_ and various clinical measures. As a possible factor explaining the weak relationships between clinical and CSMI results, the previous study noted the large differences in magnitude of vestibular stimulation, which is large in clinical vestibular tests, but small in CSMI tests. Additionally, the clinical vestibular tests in both our and their study involved ascending, vestibulo-ocular systems with short delays, while CSMI tests involve descending, vestibulo-spinal systems with substantial involvement of non-vestibular sensory information and central processing delays associated with sensory integration and motor control processes.

We also note that 14 of our 15 UV subjects showed the largest vHIT asymmetry in the lateral canals and only 5 of the 15 showed vertical canal asymmetries that would be considered abnormal (i.e., >30%). An asymmetry mainly in the lateral canal is consistent with dysfunction of the superior division of the vestibular nerve (30) that could, in theory, also affect utricular function. The vestibular contribution to standing balance control depends mainly on the vertical canals and on the utricle (31). The incomplete nature of the vestibular loss in most of the UV subjects would be thus expected to diminish the potential relationship between vHIT asymmetry measure and W_prop_ in the SS/EC condition. Nevertheless, we did see a relationship between lateral canal vHIT asymmetry and W_prop_ (Figure 6D). This relationship could occur assuming the superior vestibular nerve damage in one ear caused both lateral canal and utricular damage in that ear in proportion to the measured lateral canal vHIT asymmetry. Thus, it was the utricular damage that affected balance control leading to decreased utilization of vestibular information in the SS/EC condition. However, our evidence from vHIT tests that vestibular function in the damaged ear was only partially impaired could explain why we did not observe a clear-cut difference between the HC and UV groups in the SS/EC condition that was previously found in UV subjects with complete absence of vestibular function in one ear (12).

### Relationships of DHI scores to sensory weights

Our main expectation regarding a possible significant correlation between the DHI score and the sensory weights concerned the proprioceptive weight from the SS/EC condition where we hypothesized that for BV subjects *W*_prop_ would increase (as the availability of vestibular information decreased) with increasing DHI scores since higher scores would be associated with deteriorated vestibular function. There was a trend in this direction, but results were not significant (Table 3) and no relationship of DHI to sensory weights were observed in the SS/EO or *VS*/EO conditions. Regarding UV subjects, in the SS/EC condition, we similarly expected *W*_prop_ to increase (and vestibular use decrease) with higher DHI scores. This result was not found. However, we observed a significant correlation in the *VS*/EO condition with higher DHI values associated with larger values of *W*_vis_ (Supplementary Figure S2). This unanticipated result may be related to the manner in which some subjects compensated for vestibular dysfunction using increased reliance of visual cues that may be associated with visual complaints such as difficulty dealing with busy visual environments. We note that 4 of the 5 UV subjects with the largest W_vis_ values also had the highest DHI scores such that the high correlation was strongly influenced by results from these subjects.

### Relationship of DHI scores to vHIT and caloric measures

BV subjects with higher average vHIT gains were found to have lower (better) DHI scores consistent with greater preservation of vestibular function being associated with less perceived handicap (Figure 2A). For UV subjects, one might predict that larger vHIT asymmetries would be associated with higher (worse) DHI scores, but no relationship was found (Figure 2B). This absent relationship could be related to variation in the level of compensation among UV subjects with many having compensated quite fully and therefore not reporting symptoms despite having large vHIT asymmetries. Others who have not compensated as well or have developed comorbidities, like Persistent Postural-Perceptual Dizziness (32), could be reporting high perceived disability despite having lower vHIT asymmetries. These results highlight the heterogeneity of UV subjects making it difficult to generalize across this population and to develop discriminative measures.

### Limitations

The main limitation of this study is the small number of participants. The French speaking part of Switzerland is a small region (just over two millions inhabitants) and bilateral vestibulopathy is considered a rare condition (33). We thus anticipated recruitment would be a challenge. The limited number of subjects also limits our ability to account for age-related changes in our measures.

The second limitation of this study comes from the model we used. The CSMI model is a simplification of the human balance control system. Body mechanics are represented by an inverted pendulum body consistent of a single body segment rather than a true multi-segmental body. The model does not account for the complex, often nonlinear characteristics of sensory receptors and muscles, and for the many subsystems involved in processing and combining sensory information and generating motor commands to numerous muscle groups. Nevertheless, the overall behavior of balance control in response to low amplitude external perturbations has been shown to be well represented by a quasilinear model with the major nonlinearity represented by changes in sensory weights as a function of the magnitude of the perturbing stimulus (28) with time domain predictions of the model accounting for nearly all of the variance of the mean stimulus-evoked CoM sway (34).

Moreover, this study focused on balance control during stance and it would be important to investigate if the abnormalities we found in BV and UV subjects correspond to abnormalities found in dynamic balance control during gait in laboratory settings as well as in daily living tasks (35). The selection of the study’s participants may also have introduced some biases. Indeed, we did not consider the rehabilitation status of the patients (i.e., previous vestibular rehabilitation therapy) or their activity level in general daily life. Even though vestibular rehabilitation has shown limited long-term results for BV patients (36, 37), it has been shown to be effective for UV (38, 39). Finally, there is a known relationship between balance and cognition (40–42). Even though we excluded patients with psychiatric diseases or severe cognitive impairments, we did not assess the cognitive status of the participants. Thus, we did not identify all potential underlying factors.

Otolith organs are important contributors to vestibulo-spinal reflexes, but we only measured semicircular canal function (31). Tests of otolithic function could have contributed to the interpretation of the CSMI test results. For example, tests that characterized otolith function (e.g., ocular and cervical vestibular evoked myogenic potential, oVEMP and cVEMP, tests) may have clarified whether preserved otolith function could account for the limited differences between HC and UV subjects observed on CSMI tests. Finally, our assessments of vestibular function were based on standard measures derived from clinical tests. Advanced analysis methods may provide additional information relevant to understanding CSMI test results. One example would be an analysis that derives the variance of VOR gain measures from repeated identical head rotations (43). The variance of VOR gains is arguably indicative of the inherent noise of signals from the lateral semicircular canals in yaw-axis, earth-vertical rotation tests or from combinations of canal and otolith signals in cases where rotations are performed about a non-earth-vertical axis. VOR variability measures can also be obtained from individual head impulses from vHIT tests (44). All other factors being equal, a person with greater vestibular sensory noise is predicted to have a lower vestibular contribution to balance control compared to a person with less vestibular noise (28). If this prediction is true then an assessment of vestibular noise levels may account for some of the variance in CSMI sensory weight measures in HC, UV, and BV subjects. A second example would be to assess vestibular sensory noise levels using psychometric tests that characterize vestibular thresholds for the detection of motion with larger motion detection thresholds being associated with greater sensory noise resulting in reduced precision in a subject’s vestibular encoding of head motion (43, 45, 46).

## Conclusion

We applied the CSMI test to quantify standing balance performance and determined its reliably in distinguishing between HC, UV, and BV subjects. This test could be a useful diagnostic and rehabilitation tool as it reflects the severity of vestibular-induced functional impairment and, more importantly, could also potentially measure the efficacy of rehabilitation interventions. Indeed, objective measures of even slight improvements in vestibular function upon therapeutic interventions are desperately lacking in the field. We established a protocol providing reliable outcome measures. In particular, for patients identified as having BV deficits based on clinical vestibular tests, the *W*_prop_ measure from the SS/EC CSMI test clearly differentiated all of these subjects from all HC subjects and from nearly all subjects classified as having UV dysfunction. However, distinguishing between HC and those with UV deficits was not as successful with the poor separation likely due to the preservation of vestibular function in the damaged ear. CSMI testing revealed that vestibular-induced postural deficits increased reliance on visual cues, with this increased reliance having the potential to cause distress when visual inputs are no longer available (i.e., eyes closed or darkness) or when viewing a perturbing visual environment. Additionally, the demonstrated increased reliance on proprioceptive cues could make walking on uneven ground especially challenging. Overall, these results suggest that sensory weight measures could be used to determine if targeted rehabilitation is enhancing vestibular utilization in an individual with residual vestibular function. In the case of severe vestibular loss future methods that regenerate hair cell/nerve function or vestibular nerve activation via a vestibular implant will be likely necessary to restore functional balance control in challenging conditions where vision and proprioception are not always available or accurate.

## Data availability statement

The original contributions presented in the study are included in the article/Supplementary material, further inquiries can be directed to the corresponding author.

## Ethics statement

The studies involving humans was approved by the Commission Cantonale d’Ethique de la Recherche de la République et du Canton de Genève. The studies were conducted in accordance with the local legislation and institutional requirements. The participants provided their written informed consent to participate in this study.

## Author contributions

JC: Conceptualization, Data curation, Formal analysis, Funding acquisition, Investigation, Methodology, Writing – original draft, Writing – review & editing. J-FC: Investigation, Methodology, Writing – review & editing. AB: Investigation, Writing – review & editing. SC: Software, Writing – review & editing. MR: Investigation, Methodology, Writing – review & editing. RvdB: Writing – review & editing. RP: Conceptualization, Data curation, Formal analysis, Methodology, Software, Supervision, Validation, Writing – review & editing. NG: Funding acquisition, Methodology, Supervision, Validation, Writing – review & editing. APF: Conceptualization, Funding acquisition, Methodology, Project administration, Resources, Supervision, Validation, Writing – review & editing.
