# Supplementary material for: Postural impairments in unilateral and bilateral vestibulopathy
Source: Front Neurol. 2024 Feb 21;15:1324868. doi: 10.3389/fneur.2024.1324868 (PMC10915085; doi:10.3389/fneur.2024.1324868)
Supplement: Supplementary file 2 [file Table_1.pdf]

Supplementary Material

Postural impairments in unilateral and bilateral vestibulopathy

Julie Corre[1], Jean-François Cugnot[2], Anissa Boutabla[1], Samuel Cavuscens[1], Maurizio Ranieri[1], Raymond van de Berg[3], Robert J. Peterka[4], Nils Guinand[1], Angélica Pérez Fornos[1].

(1) Division of Otorhinolaryngology Head and Neck Surgery, Geneva University Hospitals and University of Geneva, Geneva, Switzerland
(2) Division of Clinical Neurosciences, Geneva University Hospitals, Geneva, Switzerland
(3) Division of Balance Disorders, Department of Otorhinolaryngology and Head and Neck Surgery, Maastricht University Medical Center, Maastricht, the Netherlands.
(4) National Center for Rehabilitative Auditory Research, Veterans Administration Portland Health Care System and Department of Neurology, Oregon Health & Science University, Portland, Oregon, USA

Below are detailed formulas describing the analysis methods and calculations of measures and parameters presented in the paper. The time and frequency domain analysis methods follow those described in more detail in Pintelon & Schoukens [1] and van der Kooij & Peterka [2]. Additional information related to the balance control model-based interpretation of experimental stimulus-response measures is given in Peterka [3,4], Peterka et al. [5].

RMS sway calculation

RMS (root mean squared) sway measures were calculated by averaging the CoM or head sway angles across the last 11 cycles of the stimulus, subtracting the mean from the cycle-averaged sway, calculating the mean-squared value of this zero-meaned cycle-averaged sway, and then calculating the square-root of this mean-squared value. In equation form the mean response waveform is given by:

$$\hat{r}(n) = \frac{1}{M-1}\sum_{l=2}^{M} r\left(n + lN_p\right) \qquad \text{(S1)}$$

where *n* is the sampling index, *M* is the number of stimulus cycles (nominally 12), N_p is then number of samples per stimulus cycle, and *r* is the sampled response data (either CoM sway angle or head pitch angle). $\hat{r}(n)$ is the mean response waveform that excluded the response data associated with the first stimulus cycle from the calculation of the mean.

Then the RMS value is calculated:

$$RMS = \sqrt{\sum_{n=1}^{N_p}(\hat{r}(n) - \bar{r})^2} \qquad \text{(S2)}$$

Where $\bar{r}$ is the mean value across the N_p samples of $\hat{r}(n)$.

Remnant sway calculation

The RMS value of remnant sway gives a measure that quantifies the variability of the stimulus-evoked sway response that is not accounted for by the mean value of the stimulus-evoked sway. The remnant sway calculation is performed in the frequency domain by first calculating the discrete Fourier transforms (DFTs) of the last 11 cycles of the response waveforms using the Matlab 'fft' function and calculating the average of these DFTs:

$$\hat{R}(k) = \frac{1}{M-1}\sum_{l=2}^{M} R^{[l]}\,(k) \qquad \text{(S3)}$$

Where $R^{[l]}$ is the one-sided DFT of the lth cycle of the response waveform, $\hat{R}$ is the mean DFT of the last M = 11 cycles of the response and k is the index of the frequency components of DFTs with k = 1 being the lowest frequency component of the DFT which has a value of Δf which is equal to the inverse of the 20-s single-cycle duration of stimulus (i.e., Δf = 0.05 Hz). Then the remnant power spectrum P_{rem} is calculated which is based on a variance calculation given by the squared difference of the absolute value of individual cycle DFTs from the mean DFT:

$$P_{rem}(k) = \frac{K_{sf}}{M-2} \sum_{l=2}^{M} \left| R^{[l]}(k) - \hat{R}(k) \right|^2 \quad \text{(S4)}$$

where K_{sf} is a factor that appropriately scales the power spectrum such that the area under the power spectrum is equal to the mean squared value of the signal. Specifically, K_{sf} is the inverse of the product of two times the time series sampling rate times the number of samples per stimulus cycle. Finally, the remnant RMS value is calculated by taking the square root of the summed value of the area under the remnant power spectrum:

$$RMS_{rem} = \sqrt{\Delta f \cdot \sum_{k=1}^{kmax} P_{rem}(k)} \quad \text{(S5)}$$

With $kmax$ = 100 (corresponding to 5 Hz) being the highest frequency component index that was used in the summation.

Central Sensorimotor Integration (CSMI) analysis

The experimental frequency response function (FRF) provides a non-parametric, frequency-domain characterization of the dynamic properties of the balance control system. The experimental FRF was calculated by dividing the cycle-averaged DFT of the sway response by the cycle-averaged DFT of the stimulus:

$$H_{exp}(k) = \hat{R}(k)/\hat{S}(k) \quad \text{(S6)}$$

Where $\hat{R}(k)$ is defined in equation S3 and $\hat{S}(k)$ is the average stimulus DFT defined in a similar manner. Smoothing was applied to H_{exp} by averaging adjacent frequency points to reduce the variance of H_{exp} and to provide H_{exp} measures that were approximately equally spaced on a logarithmic frequency scale. Examples of H_{exp} calculations are shown in **Figure 1C** of the paper for a healthy control and bilateral vestibular loss subject.

The non-parametric experimental FRFs that characterized the dynamics of CoM responses to surface tilt or visual tilt stimuli were used to estimate the values of functionally relevant parameters of the balance control system by adjusting the parameters of a balance control model.

The block diagram of the CSMI model in **Figure 1B** of the paper can be expressed as a differential equation that determines the body sway angle, *CoM*, relative to Earth vertical as a function of the support surface, *SS*, stimulus and/or the visual scene, *VS*, stimulus under steady-state conditions when all transient responses that occur at stimulus initiation have decayed to negligible amounts. When all the dynamic elements of the model (which include the inverted pendulum body, *B*, the 'motor activation' component, *MA*, 'torque feedback', *TF*, and 'time delay', *TD*) are expressed in the Laplace domain, the equations relating *CoM* to *SS* (in both eyes open and closed conditions) and/or *VS* can be solved algebraically to define 'transfer functions', *H*, that express the dynamic relationship between the stimulus and the body sway response:

$$H_{SS\ to\ CoM} = \frac{W_{prop} \cdot MA \cdot TD \cdot B}{1 - TF \cdot MA \cdot TD + MA \cdot TD \cdot B} \quad \text{(S7)}$$

$$H_{VS\ to\ CoM} = \frac{W_{vis} \cdot MA \cdot TD \cdot B}{1 - TF \cdot MA \cdot TD + MA \cdot TD \cdot B} \quad \text{(S8)}$$

With:

$$B = \frac{1}{J \cdot s^2 - mgh} \quad \text{(S9)}$$

$$MA = K_p + K_d \cdot s \quad \text{(S10)}$$

$$TD = e^{-T_d \cdot s} \quad \text{(S11)}$$

$$TF = \frac{K_t}{s} \quad \text{(S12)}$$

where 's' is the Laplace variable. Substituting S9 – S12 into S7 and S8 and setting $s = j2\pi f$, where j is the imaginary number $\sqrt{-1}$, allows for the calculation of the model H values as a function of the sinusoidal stimulus frequency, f. All transfer function equations assume that the sum of all sensory weights contributing to balance, in a given condition, sum to 1 meaning the value of a sensory weight represents the relative contribution of a sensory system to balance control. For example, in the eyes open surface stimulus condition proprioception, visual, and vestibular cues are the contributors to balance control. The curve fitting procedure will estimate the value of W_{prop} for this condition and then the vestibular plus visual contribution is given by $W_{vest} + W_{vis} = 1 - W_{prop}$.

The value of H, at any particular frequency, f, is a complex number that can be expressed in terms of a 'magnitude function' $|H(j2\pi f)|$ equal to the square root of the sum of squared values of the real and imaginary components of H, and 'phase function' $\angle H(j2\pi f)$ equal to the arc tangent of the imaginary divided by the real components of H. The transfer function magnitude is also referred to as the system 'gain function' since it represents the body sway response magnitude normalized by the magnitude of the stimulus at each frequency value.

The free parameters were adjusted to optimally account for the experimental FRF, H_{exp}, derived from the experimental body sway responses to the pseudorandom stimulus (equation S6) using Matlab Optimization Toolbox function 'fmincon'. The free parameters include the sensory weight (W), motor activation 'stiffness' parameter (K_p) and 'damping' parameter (K_d), time delay (T_d), and torque feedback (K_t). The body moment of inertia about the ankle joint, J, body mass, m, (excluding the feet), and body center-of-mass height above the ankle joints, h, are derived from direct measurement of body mass and based on anthropometric body measures [6], and g is the gravity constant. Examples of experimental FRFs and calculated FRFs derived from transfer function equations with optimally adjusted parameters are shown in **Figure 1C** in the paper.

Supplementary Table S1: RMS values of stimulus-evoked CoM and head sway, remnant CoM and head sway for HC, UV, and BV groups and post-hoc comparisons between groups.

		**Groups**						ANOVA*/Kruskal-Wallis**	Post Hoc Sig.			Hedges' G value		
		**HC**		**UV**		**BV**								
		Mean	±SD	Mean	±SD	Mean	±SD	p value	HS vs BV	HS vs UV	UV vs BV	HS vs BV	HS vs UV	UV vs BV
**CSMI condition**	**Measure**													
**SS / EO**														
	N completing condition	20		15		17								
	**Stimulus-evoked CoM sway**	0.538	0.158	0.625	0.272	0.652	0.201	0.233*	N/A	N/A	N/A	0.625	0.395	0.114
	**Stimulus-evoked Head sway**	0.433	0.696	0.587	0.470	0.499	0.299	**0.027****	0.114	**0.043**	1	0.117	0.247	0.222
	**Remnant CoM sway**	0.248	0.123	0.470	0.435	0.264	0.117	**0.039****	1	**0.043**	0.214	0.132	0.727	0.649
	**Remnant Head sway**	0.796	1.355	1.552	1.712	1.039	1.223	**0.013****	0.182	**0.012**	0.925	0.184	0.487	0.340
**SS / EC**														
	N completing condition	20		15		17								
	**Stimulus-evoked CoM sway**	0.760	0.154	0.912	0.263	1.094	0.198	**<0.001****	**<0.001**	0.067	0.060	1.859	0.713	0.768
	**Stimulus-evoked Head sway**	0.768	0.289	1.415	1.381	1.250	0.389	**<0.001****	**<0.001**	**0.011**	0.650	1.392	0.683	0.164
	**Remnant CoM sway**	0.279	0.122	0.500	0.292	0.590	0.357	**<0.001****	**<0.001**	**<0.001**	0.978	1.18	1.022	0.778
	**Remnant Head sway**	0.860	0.428	1.919	3.568	1.603	0.835	**< 0.001****	**<0.001**	0.808	**0.048**	1.125	0.441	0.122
**VS / EO**														
	N completing condition	20		15		17								
	**Stimulus-evoked CoM sway**	0.131	0.060	0.216	0.112	0.315	0.142	**<0.001****	**<0.001**	0.091	0.157	1.708	0.959	0.752
	**Stimulus-evoked Head sway**	0.297	0.170	0.465	0.394	0.541	0.199	**0.001****	**<0.001**	0.275	0.225	1.301	0.572	0.243
	**Remnant CoM sway**	0.223	0.137	0.339	0.306	0.264	0.105	0.131**	NA	NA	NA	0.323	0.502	0.327
	**Remnant Head sway**	0.506	0.268	1.050	1.437	0.985	0.726	**0.025****	**0.020**	0.788	0.448	0.885	0.555	0.466

Note: SS/EO = surface stimulus eyes open, SS/EC = surface stimulus eyes closed, VS/EO = visual stimulus eyes open, SD = standard deviation. All sway measures have units of degrees. p-values indicate results of a One-Way Analysis of Variance (ANOVA)* or Kruskal-Wallis One-Way Analysis of Variance**. Post hoc analysis is either Dunn's (following Kruskal-Wallis) or Holm-Sidak (following ANOVA) method. In addition to the p value, we also calculated the Hedge's G value, which represents an effects size measure, measuring the difference between means relative to the standard deviation. Bolded outcomes indicate a significant group difference.

Supplementary Table S2: Central Sensorimotor Integration (CSMI) test model-derived parameters for HS, UV, and BV groups and post-hoc comparisons between groups.

		Groups						ANOVA*/ Kruskal-Wallis**	Post Hoc Sig.			Hedge'G value		
		**HS**		**UV**		**BV**								
		Mean	±SD	Mean	±SD	Mean	±SD	p value	HS vs BV	HS vs UV	UV vs BV	HS vs BV	HS vs UV	UV vs BV
**CSMI condition**	**Parameter**													
**SS / EO**														
	n completing condition	20		15		17								
	**Proprioceptive weight**	0.343	0.053	0.355	0.069	0.426	0.083	**0.001***	**0.002**	0.616	**0.01**	1.193	0.191	0.910
	**Vestibular + visual weight**	0.657	0.053	0.629	0.075	0.574	0.083	**0.001***	**0.002**	0.616	**0.01**	1.193	0.191	0.910
	**Time delay (s)**	0.134	0.023	0.147	0.031	0.160	0.012	**0.006***	**0.004**	0.216	0.116	1.331	0.460	0.555
	Normalized stiffness	1.693	0.281	1.658	0.463	1.568	0.147	0.42**	N/A	N/A	N/A	0.534	0.093	0.263
	Normalized damping	0.545	0.076	0.526	0.104	0.564	0.076	0.466*	N/A	N/A	N/A	0.24	0.206	0.405
**SS / EC**														
	n completing condition	20		15		17								
	**Proprioceptive weight**	0.565	0.062	0.611	0.062	0.917	0.109	**<0.001****	**<0.001**	0.570	**<0.001**	3.967	0.728	3.306
	**Vestibular + vestibular weight**	0.435	0.062	0.381	0.065	0.083	0.109	**<0.001****	**<0.001**	0.570	**<0.001**	3.967	0.728	3.306
	**Time delay (s)**	0.148	0.014	0.157	0.026	0.164	0.018	**0.040***	**0.035**	0.333	0.261	1.021	0.440	0.343
	**Normalized stiffness**	1.719	0.238	1.572	0.218	1.603	0.138	0.068**	N/A	N/A	N/A	0.573	0.627	0.168
	Normalized damping	0.573	0.050	0.545	0.094	0.570	0.049	0.784**	N/A	N/A	N/A	0.049	0.382	0.340
**VS / EO**														
	n completing condition	20		15		17								
	**Visual weight**	0.078	0.037	0.120	0.042	0.271	0.118	**< 0.001****	**<0.001**	0.157	**0.007**	2.259	0.963	1.674
	**Proprioceptive + vestibular weight**	0.922	0.037	0.880	0.046	0.729	0.118	**<0.001****	**<0.001**	0.157	**0.007**	2.259	0.963	1.674
	Time delay (s)	0.187	0.024	0.196	0.037	0.176	0.022	0.131*	N/A	N/A	N/A	0.462	0.299	0.656
	**Normalized stiffness**	1.261	0.094	1.278	0.128	1.352	0.110	**0.02****	**0.027**	1	0.089	0.876	0.145	0.616
	Normalized damping	0.494	0.052	0.493	0.068	0.523	0.047	0.233*	N/A	N/A	N/A	0.576	0.016	0.512

Note: SS/EO = surface stimulus eyes open, SS/EC = surface stimulus eyes closed, VS/EO = visual stimulus eyes open, SD = standard deviation, s = seconds. p-values indicate results of a One-Way Analysis of Variance (ANOVA)* or Kruskal-Wallis One-Way Analysis of Variance**. Post hoc analysis is either Dunn's (following Kruskal-Wallis) or Holm-Sidak (following ANOVA) method. In addition to the p value, we also calculated the Hedge's G value, which represents an effects size measure, measuring the difference between means relative to the standard deviation. Bolded outcomes indicate a significant group difference.

Supplementary Table S3: Mean and SD values of CSMI test measures and post-hoc comparisons between test conditions.

	Condition									
	SS/EO		SS/EC		VS/EO		**Kruskall-Wallis**		**Post-Hoc Sig.**	
**Measure**	Mean	± SD	Mean	± SD	Mean	± SD	p value	SS/EO vs SS/EC	SS/EO vs VS/EO	SS/EC vs VS/EO
**Stimulus-evoked CoM sway**	0.6	0.212	0.913	0.245	0.216	0.131	**<0.001**	**<0.001**	**<0.001**	**<0.001**
**Stimulus-evoked Head sway**	0.499	0.523	1.112	0.826	0.425	0.278	**<0.001**	**<0.001**	0.964	**<0.001**
**Remnant CoM sway**	0.317	0.268	0.563	0.364	0.27	0.196	**<0.001**	**<0.001**	0.575	**<0.001**
**Remnant Head sway**	1.094	1.434	1.408	1.997	0.82	0.907	**<0.001**	0.001	0.068	**<0.001**
**Time delay**	0.146	0.025	0.156	0.02	0.186	0.029	**<0.001**	0.217	**<0.001**	**<0.001**
**Normalized stiffness**	1.642	0.313	1.639	0.211	1.296	0.115	**<0.001**	0.758	**<0.001**	**<0.001**
**Normalized damping**	0.546	0.085	0.564	0.065	0.504	0.055	**<0.001**	0.279	**0.008**	**<0.001**
							**Mann-Whitney**			
							p value			
							SS/EO vs SS/EC			
**Proprioceptive weight**	0.374	0.077	0.694	0.177	0.152	0.112	**<0.001**			

Note: Data were combined across HC, UV, and BV groups for these comparisons. SS/EO = surface stimulus eyes open, SS/EC = surface stimulus eyes closed, VS/EO = visual stimulus eyes open, SD = standard deviation. p-values indicate results of either a Kruskal-Wallis One-Way Analysis of Variance or a Mann-Whitney test. All Pairwise Multiple Comparison Procedures used a Tukey test. Bolded outcomes indicate a significant group difference.

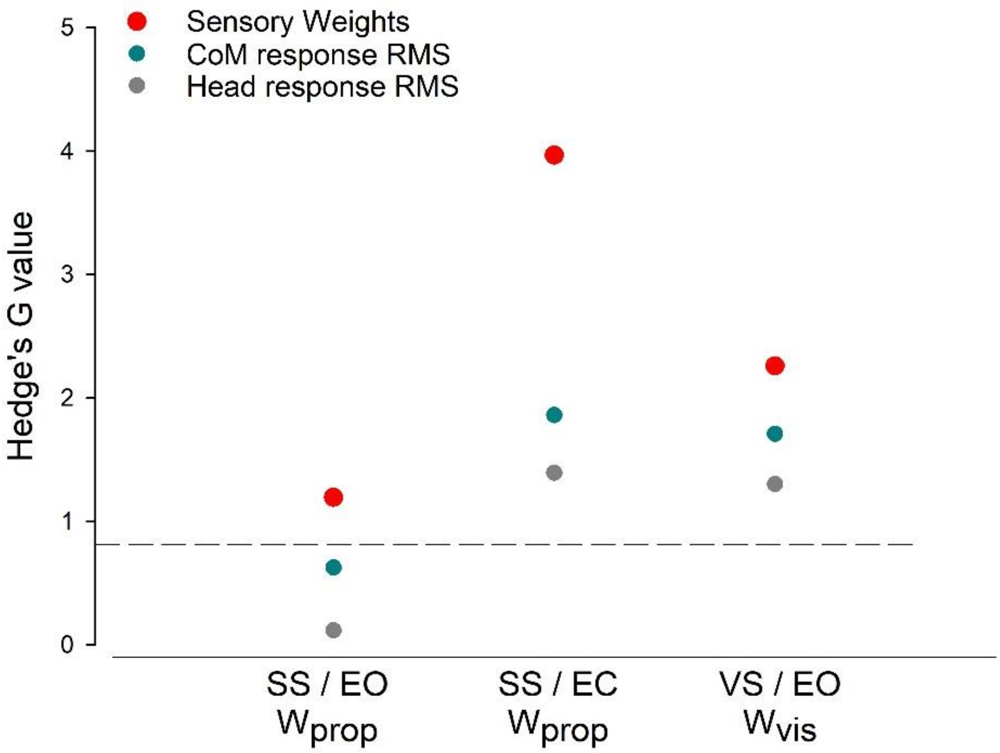

Supplementary Figure S1*. Hedge's G values for CoM stimulus-evoked RMS sway, Head motion RMS sway, and Sensory weights across the three conditions comparing results from bilateral vestibular deficit and healthy control groups. Hedge's G values greater than 0.8 (dashed line) are considered to be large effect sizes.*

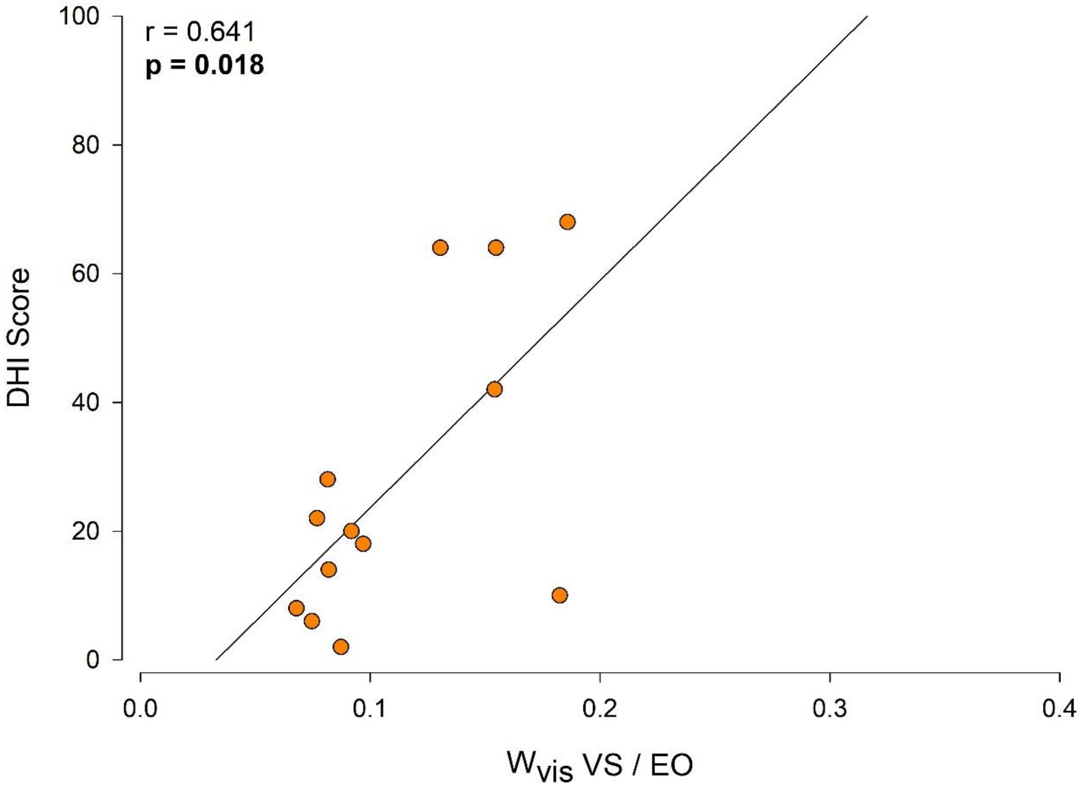

***Supplementary Figure S2**. Relationship of Dizziness Handicap Inventory (DHI) to visual weight measures (W_{vis}) in unilateral vestibular loss subjects from CSMI visual stimulus condition. Self-perceived handicap is considered moderate for DHI scores between 30 and 60, and severe for DHI scores above 60. The Pearson product-moment correlation coefficient (r) and associated p values are shown.*

References

1. Pintelon R, Schoukens J. System identification: a frequency domain approach: John Wiley & Sons; 2012.
2. van der Kooij H, Peterka RJ. Non-linear stimulus-response behavior of the human stance control system is predicted by optimization of a system with sensory and motor noise. J Comput Neurosci. 2011;30(3):759-78.
3. Peterka RJ. Sensorimotor integration in human postural control. J Neurophysiol. 2002;88(3):1097-118.
4. Peterka RJ. Simplifying the complexities of maintaining balance. IEEE Engineering in Medicine and Biology Magazine. 2003;22(2):63-8.
5. Peterka RJ, Murchison CF, Parrington L, Fino PC, King LA. Implementation of a Central Sensorimotor Integration Test for Characterization of Human Balance Control During Stance. Front Neurol. 2018;9:1045.
6. Winter DA. Biomechanics and motor control of human movement: John Wiley & Sons; 2009.
